# A Physical Activity Clinical Practice Guideline for People With Moderate to Severe Traumatic Brain Injury

**DOI:** 10.1002/gin2.70035

**Published:** 2025-08-14

**Authors:** Leanne Hassett, Liam Johnson, Gavin Williams, Rhys Ashpole, Adrian Bauman, Catherine Carty, Sakina Chagpar, Kelly Clanchy, Abby Haynes, Kate Heine, Zachary Munn, Anthony Okely, Nick Rushworth, Adam Scheinberg, Catherine Sherrington, Grahame Simpson, Anne Tiedemann, Sean Tweedy, Gabrielle Vassallo, Belinda Wang, Kerry West, Luke Wolfenden, the BRIDGES Guideline Development Group members, Pien Alferink, Rhys Ashpole, Adrian Bauman, Olivia Beattie, Francesca Brady, Catherine Carty, Sakina Chagpar, Daniel Cheung, Kelly Clanchy, Alexandra Edmondson, Joanne Glinsky, Leanne Hassett, Abby Haynes, Kate Heine, Sonia Hoppe, Liam Johnson, Anthony Mamo, Peter Mayhew, Zachary Munn, Anthony Okely, Nick Rushworth, Sania Salim, Benjamin Sammut, Adam Scheinberg, Catherine Sherrington, Grahame Simpson, Anne Tiedemann, Sean Tweedy, Gabrielle Vassallo, Sarah Veli‐Gold, Belinda Wang, Nicholas Waters, Kerry West, Bhavini Whiteside, Gavin Williams, Luke Wolfenden, Ella Bracone, Domenic Denichilo, Aleksandra Gozt, Julie, Kieren Witts

**Affiliations:** ^1^ Institute for Musculoskeletal Health Sydney Local Health District Sydney New South Wales Australia; ^2^ Sydney School of Health Sciences, Faculty of Medicine and Health The University of Sydney Sydney New South Wales Australia; ^3^ Western Sydney Local Health District Sydney New South Wales Australia; ^4^ School of Health Sciences, Faculty of Medicine, Dentistry and Health Sciences University of Melbourne Melbourne Victoria Australia; ^5^ Physiotherapy Department Epworth HealthCare Melbourne Victoria Australia; ^6^ School of Behavioural and Health Sciences, Faculty of Health Sciences Australian Catholic University Melbourne Victoria Australia; ^7^ icare NSW Sydney New South Wales Australia; ^8^ Sydney School of Public Health, Faculty of Medicine and Health The University of Sydney Sydney New South Wales Australia; ^9^ Munster Technological University Tralee Kerry Ireland; ^10^ School of Health Sciences and Social Work, Griffith Health Griffith University Gold Coast New South Wales Australia; ^11^ The Hopkins Centre Griffith University Nathan Queensland Australia; ^12^ Heads Together for Brain Injury Melbourne Victoria Australia; ^13^ Health Evidence Synthesis, Recommendations and Impact (HESRI), School of Public Health, Faculty of Health and Medical Sciences University of Adelaide Adelaide South Australia Australia; ^14^ School of Health and Society University of Wollongong Wollongong New South Wales Australia; ^15^ Brain Injury Australia Sydney New South Wales Australia; ^16^ Murdoch Children's Research Institute Melbourne Victoria Australia; ^17^ Liverpool Brain Injury Rehabilitation Unit South Western Sydney Local Health District Sydney New South Wales Australia; ^18^ John Walsh Centre for Rehabilitation Research, Kolling Institute Northern Sydney Local Health District and The University of Sydney Sydney New South Wales Australia; ^19^ School of Human Movement and Nutrition Sciences, Faculty of Health and Behavioural Sciences University of Queensland Brisbane Queensland Australia; ^20^ Health and Wellbeing Centre for Research Innovation The University of Queensland St Lucia Queensland Australia; ^21^ Queensland Centre for Olympic and Paralympic Studies The University of Queensland St Lucia Queensland Australia; ^22^ Consumer representative Sydney New South Wales Australia; ^23^ Physiotherapy Department The Children's Hospital at Westmead Sydney New South Wales Australia; ^24^ Hunter New England Local Health District University of Newcastle Newcastle New South Wales Australia; ^25^ The Royal Children's Hospital Melbourne Victoria Australia; ^26^ Person with Lived Experience New South Wales Australia; ^27^ School of Health Sciences and Social Work, Griffith Health Griffith University, Gold Coast, Australia; The Hopkins Centre, Griffith University Nathan Queensland Australia; ^28^ School of Health and Rehabilitation Sciences The University of Queensland Brisbane Queensland Australia; ^29^ Person with Lived Experience Sydney New South Wales Australia; ^30^ Person with Lived Experience Perth Western Australia Australia; ^31^ Person with Lived Experience Melbourne Victoria Australia; ^32^ Central Australia region, NT Health Alice Springs Northern Territory Australia; ^33^ Connectivity Traumatic Brain Injury Australia Perth Western Australia Australia; ^34^ Family members of Person with Lived Experience Victoria Australia

**Keywords:** adult, GRADE ADOLOPMENT, guideline, paediatric, physical activity, rehabilitation, traumatic brain injury

## Abstract

**Introduction:**

In 2020 the World Health Organization (WHO) released the first international public health guideline on physical activity for people with disability. However, the evidence informing the guideline was not specific to people with traumatic brain injury (TBI), nor did it provide guidance for health professionals to promote and deliver physical activity in rehabilitation. We aimed to develop an Australian Physical Activity Clinical Practice Guideline for people with moderate to severe TBI (msTBI).

**Questions:**

This guideline sought to answer the following question: ‘*Should **[physical activity intervention]** compared to control be used for **[people with]** msTBI?’* The question was adapted to five physical activity interventions (structured aerobic exercise, muscle strengthening, gait/balance/functional exercise, sport and physical recreation, and promotion of physical activity) and two populations (children and adolescents; adults and older adults).

**Methods:**

We used the Grading of Recommendations Assessment, Development and Evaluation (GRADE) ADOLOPMENT approach to determine whether to ‘adapt’ or ‘adopt’ the WHO guideline or develop *de novo* recommendations. We established guideline steering, leadership and development groups, conducted a rapid review to identify direct evidence in msTBI, and reviewed guidelines in other relevant health conditions (i.e., stroke, cerebral palsy) to identify indirect evidence. To address evidence gaps and inform implementation considerations, we conducted an audit of brain injury services in Australia and qualitative consultations with key interest‐holders, including people with msTBI.

**Recommendations:**

The guideline incorporates 10 *de novo* recommendations for people with msTBI for the delivery and promotion of physical activity across the rehabilitation continuum of care. The guideline includes good practice and precautionary points, and subgroup considerations to guide usability and implementation. Data from the clinical audit and interest‐holder focus groups indicated feasibility and acceptability of physical activity interventions. The guideline seeks to support health professionals' clinical decision‐making and increase uptake of physical activity by people with msTBI.

## Summary

- This is the first Clinical Practice Guideline to address the unique physical activity needs of people with moderate to severe traumatic brain injury (msTBI), a group excluded from previous international physical activity guidelines. It fills a critical gap by recognising the specific challenges and stages of recovery in this population.
- The Exploration Preparation Implementation Sustainment (EPIS) framework and GRADE‐ADOLOPMENT process were used to integrate a rapid systematic review, national audit, and interest‐holder consultations to ensure the recommendations are relevant, credible, and grounded in both research and real‐world practice.
- The guideline provides health professionals with 10 evidence‐informed recommendations, alongside good and precautionary practice points, to guide safe and effective prescription and promotion of physical activity for children, adolescents, adults and older adults with msTBI in a range of settings and across the rehabilitation continuum.
- The Guideline was approved by the National Health and Medical Research Council (NHMRC) of Australia. This signals the guideline meets national standards for trustworthiness, making it a credible and practical resource for health professionals supporting people with msTBI in the Australian health care setting.
- Evidence gaps remain; there remains limited evidence in subpopulations including Aboriginal and Torres Strait Islander people, people from Culturally and Linguistically Diverse backgrounds, older adults, children, those with high support needs, and those living in regional and rural Australia. Further research is needed to strengthen the recommendations and develop practical tools for implementation.

## 1 Introduction and Scope

Physical activity is highly beneficial, both to the individual who participates in the activity and to society more broadly [1, 2]. Despite these benefits and the existence of global physical activity targets and policies to increase population‐level physical activity, physical inactivity remains a leading global health challenge, with little improvement over the past 25 years [3, 4]. Adults and children with disabilities are twice as likely not to meet recommended physical activity levels compared to those without disability [5]. Urgent action is required to enable people with disability to gain the health and social benefits of being physically active.

Traumatic brain injury (TBI) is a leading cause of long‐term disability with physical, cognitive and/or behavioural impairments, and can occur any time along the lifespan [6]. Moderate and severe TBI (msTBI) makes up a small proportion of the total incidence (23 moderate TBI/100,000 and 13 severe TBI/100,000), however msTBI is estimated to account for most health‐related costs [7] and people with msTBI are at high risk of developing chronic health conditions [8]. While health conditions such as stroke are more common, msTBI primarily affects people during their most economically productive years, and the effects are lifelong [9]. Consequently, the economic and social costs of msTBI are disproportionately high.

People with msTBI are highly inactive [11]. Physical inactivity from prolonged bed rest is extensive and extreme in the first weeks to months after msTBI, and this extends into inpatient rehabilitation [12, 13, 14]. At the time of discharge from hospital, most people with msTBI are independent in their mobility [15], yet both adults and children with msTBI continue to be more physically inactive than age‐matched peers in the long‐term [16].

The 2020 World Health Organization (WHO) Physical Activity and Sedentary Behaviour public health guideline update included, for the first‐time, specific recommendations for adults and children with disability [17]. The recommendations were informed by direct evidence from physical activity interventions (excluding rehabilitation) relating to eight health conditions (not including TBI), as well as indirect evidence from general age‐specific populations. Physical activity clinical practice guidelines exist for other health conditions (e.g., spinal cord injury [18]); however, no physical activity clinical practice guideline currently exists to guide health professionals working with people with msTBI. Given this gap, there is a pressing need for the rigorous development of a physical activity clinical practice guideline that considers the benefits, risks, values, and preferences of people with msTBI.

The Australian TBI Mission is a long‐term investment in research to improve patient recovery after brain injury [19]. The development of the Australian Physical Activity Clinical Practice Guideline for people with msTBI was funded by the Medical Research Future Fund TBI Mission in 2020. The aim of the guideline is to support the promotion and prescription of high‐value and consistent evidence‐based physical activity interventions across the continuum of care. The guideline is targeted towards health professionals working with people with msTBI, including children and adolescents, and adults and older adults, to improve their engagement in, and access to, physical activity. The guideline is applicable across the rehabilitation continuum of care, in settings likely to include inpatient, transition and outpatient hospital settings, community settings (e.g., fitness centres), homes and schools (Table 1).

**Table 1:** Australian Physical Activity Clinical Practice Guideline for people with moderate to severe traumatic brain injury—Summary of recommendations.

Recommendation	Strength and certainty of evidence
For adults and older adults after moderate to severe traumatic brain injury, we suggest regular structured aerobic exercise that is individually‐tailored and across the continuum of care.	*Conditional recommendation FOR, low certainty evidence*.
For children and adolescents after moderate to severe traumatic brain injury, we suggest regular energetic play and/or exercise that is individually tailored and across the continuum of care.	*Conditional recommendation FOR, very low certainty evidence*.
For adults and older adults after moderate to severe traumatic brain injury, we recommend individually‐tailored muscle strengthening exercise, including ballistic training, across the continuum of care.	*Strong recommendation FOR, moderate certainty evidence*.
For children and adolescents after moderate to severe traumatic brain injury, we suggest regular muscle strengthening play and/or exercise that is individually‐tailored and across the continuum of care.	*Conditional recommendation FOR*.*
For adults and older adults after moderate to severe traumatic brain injury, we recommend task‐specific mobility training across the continuum of care.	*Strong recommendation FOR, moderate certainty evidence*.
For children and adolescents after moderate to severe traumatic brain injury, we suggest task‐specific mobility training across the continuum of care.	*Conditional recommendation FOR, very low certainty evidence*.
For adults and older adults after moderate to severe traumatic brain injury, we suggest participation in sport and physical recreation across the continuum of care considering their personal preference and capability.	*Conditional recommendation FOR, very low certainty evidence*.
For children and adolescents after moderate to severe traumatic brain injury, we suggest participation in sport and physical recreation across the continuum of care considering their personal preference and capability.	*Conditional recommendation FOR*.*
For adults and older adults after moderate to severe traumatic brain injury, we suggest the promotion of physical activity across the continuum of care.	*Conditional recommendation FOR, low certainty evidence*.
For children and adolescents after moderate to severe traumatic brain injury, we suggest the promotion of physical activity across the continuum of care.	*Conditional recommendation FOR*.*

*Note:* The direction of a recommendation is expressed as FOR an intervention, AGAINST an intervention or no recommendation [10]. The strength of a recommendation for or against an intervention was expressed as Strong or Conditional. Unshaded rows are for adults and older adults and the shaded rows are for children and adolescents.

* In the absence of direct evidence, certainty of evidence could not be assessed.

## 2 Methods

The Exploration, Preparation, Implementation, Sustainment (EPIS) framework [20] was used as an overarching implementation process framework to ensure the guideline was developed with future implementation in mind. In developing the guideline, we planned on using the Grading of Recommendations Assessment, Development and Evaluation (GRADE) ADOLOPMENT approach [21]. See Schünemann et al. [22] and Okely et al. [23] for a detailed methodology of this approach.

### 2.1 Panel Composition

Guideline Steering, Leadership, and Development Groups were established with responsibility for the development of the guideline. The composition and purpose of the three groups is detailed in Appendix S3. Members of the groups were identified by the guideline chair and co‐chair, L.H. and L.J., respectively, and either approached directly by L.H. or appointed by their organisation. The Guideline Steering Group was tasked with conducting the research and administrative tasks associated with organising and completing the guideline. The Guideline Steering Group reported to the Guideline Leadership Group. All members of the Guideline Leadership Group were involved in developing the scope and processes for each committee. Only members of the Guideline Development Group were involved in deciding on the recommendations, including their wording, contained within the clinical practice guideline.

### 2.2 Questions and Outcomes

The clinical questions addressed in this guideline are presented in the PICO format (i.e., **P**articipant, **I**ntervention, **C**omparison, and **O**utcome). This guideline sought to answer the following question: *‘Should **[physical activity intervention]** compared to control be used for **[people with]** msTBI?*’ The question was adapted to five physical activity interventions (structured aerobic exercise, muscle strengthening, gait/balance/functional exercise, sport and physical recreation, and promotion of physical activity) and two populations (children and adolescents; adults and older adults). The clinical questions were drafted by the Guideline Steering Group and presented to the Guideline Leadership Group for discussion and approval of their adoption before commencing the systematic review.

A range of outcomes were identified from those evaluated in the development of the WHO physical activity and sedentary behaviour guideline for people with disability [17]. The outcomes of interest were selected based on clinical questions and focused on impairment, activity and participation levels within the International Classification of Functioning, Disability and Health framework [24]. Additional outcomes considered by the Guideline Leadership Group of importance for people with msTBI were also included. From a list of 15 outcomes, the Guideline Leadership Group members ranked each outcome in terms of level of importance to a person with msTBI for each question. Only outcomes ranked critical (score 7–9/9) or important (score 4–6/9) for clinical decision‐making were included in the final list of outcomes, with a maximum of seven for each question [25] (see Appendix S4 for the critical and important outcomes for each clinical question).

### 2.3 Identification of Credible Existing Guidelines

We identified and prioritised potentially relevant, credible existing guidelines from which to adapt, or adopt, to develop the clinical practice guideline. The guidelines were identified via a customised search of online databases and expert consultation of potentially relevant published guidelines based on the overall PICO for this guideline. The following criteria were applied to determine their selection: (1) published in the past 10 years; (2) addressed PICO clinical questions; (3) followed the GRADE process, including a risk‐of‐bias (RoB) assessment; (4) allowed for updating; and 5. included existing and accessible GRADE and summary of findings tables.

The identified guidelines were also rated on whether they included information on guideline implementation and dissemination, and whether they included benefits and harms assessments for patient‐important outcomes. A total of 13 guidelines were rated (Appendix S5). The Steering Group then submitted their recommendations to the Guideline Leadership Group for approval of the guideline/s to be adapted or adopted based on the following criteria: (1) quality of guideline; (2) appropriate scope/applicability for Australia; (3) priority topic for Australia; and (4) PICO questions for the systematic review are relevant to the topic and questions.

Following discussions by the Guideline Leadership Group, the Australian stroke guideline [26], WHO physical activity and sedentary behaviour guideline for people with disability [17], and international cerebral palsy guideline [27] were identified as being credible existing guidelines and could provide indirect evidence where there was no/limited evidence in msTBI. However, given their limited applicability to the condition (i.e., people with msTBI) and setting (i.e., rehabilitation was not considered in the WHO guideline), the Guideline Leadership Group decided the creation of *de novo* recommendations following traditional GRADE approaches [28, 29]) was more appropriate than the adaptation or adoption of existing guidelines.

The Guideline Leadership Group also decided to update the search strategy used by the WHO guideline [17] to examine the association between physical activity and health‐related outcomes among people with stroke given the overlap in impairments experienced by people with stroke and people with msTBI. Thirty‐five systematic reviews were identified from the updated WHO search. A member of the Guideline Steering Group used the AMSTAR 2 (A MeaSurement Tool to Assess systematic Reviews) instrument [30] to critically appraise the reviews, with two rated as having high [31] or moderate [32] credibility and considered as part of indirect evidence to inform guideline development. Appendix S6 presents the AMSTAR 2 ratings for each review considered for inclusion and the evidence profile and extracted data for each included review updated from the original WHO search strategy.

### 2.4 Approach for Moving From Evidence to Recommendation: Creating GRADE Evidence to Decision Frameworks for Each Clinical Question

#### 2.4.1 Determine Availability, Completeness and Currency of Information About Evidence to Decision Criteria

Given the lack of direct evidence that could be acquired from current credible guidelines, the Guideline Leadership Group decided on an update to a recent rapid systematic review [33] as the best source of direct evidence to inform the development of *de novo* recommendations.

The following is a brief overview of the systematic review literature search, evidence eligibility, and evidence synthesis methods. Further details are provided in Appendix S7 and can be found in the Guideline Technical Report.

#### 2.4.2 Literature Searches and Evidence Eligibility

A systematic review was conducted to inform the Evidence to Decision (EtD) criteria and recommendations. The PICO framework was used to define the scope of the review (see Table 2).

**Table 2:** Systematic review eligibility criteria.

PICO Element + study design	Eligibility criteria
Population	People of all ages with msTBI
Intervention	A physical activity intervention, including structured aerobic exercise, muscle strengthening, gait/balance/functional exercise, sport and physical recreation, and promotion of physical activity
Comparison	Usual care, a physical activity intervention with different parameters (i.e., dose, setting, or supervision), a nonphysical activity intervention, or no intervention
Outcome	Physical function (i.e., cardiorespiratory fitness, mobility, balance, muscle strength, body composition, walking capacity, fatigue), cognition, quality of life, comorbidities and mortality, mood, participation, physical activity, behaviour change, pain, and social connection
Study design	Published RCTs (including cross‐over RCTs) and NRSIs

Abbreviations: msTBI, moderate to severe traumatic brain injury; NRSI, Non‐randomised studies of interventions; PICO, Population, Intervention, Comparison, Outcome; RCTs, Randomised controlled trials.

The following electronic databases were searched from inception to December 24th, 2022, to identify reports of relevant studies: Ovid MEDLINE, Ovid CENTRAL, and EBSCO SPORTDiscus. Search terms for randomised controlled trials (RCTs), systematic reviews, and non‐randomised studies of interventions (NRSIs), were combined with search terms for physical activity and TBI. See Appendix S8 for full search strategies.

**Figure 1:**
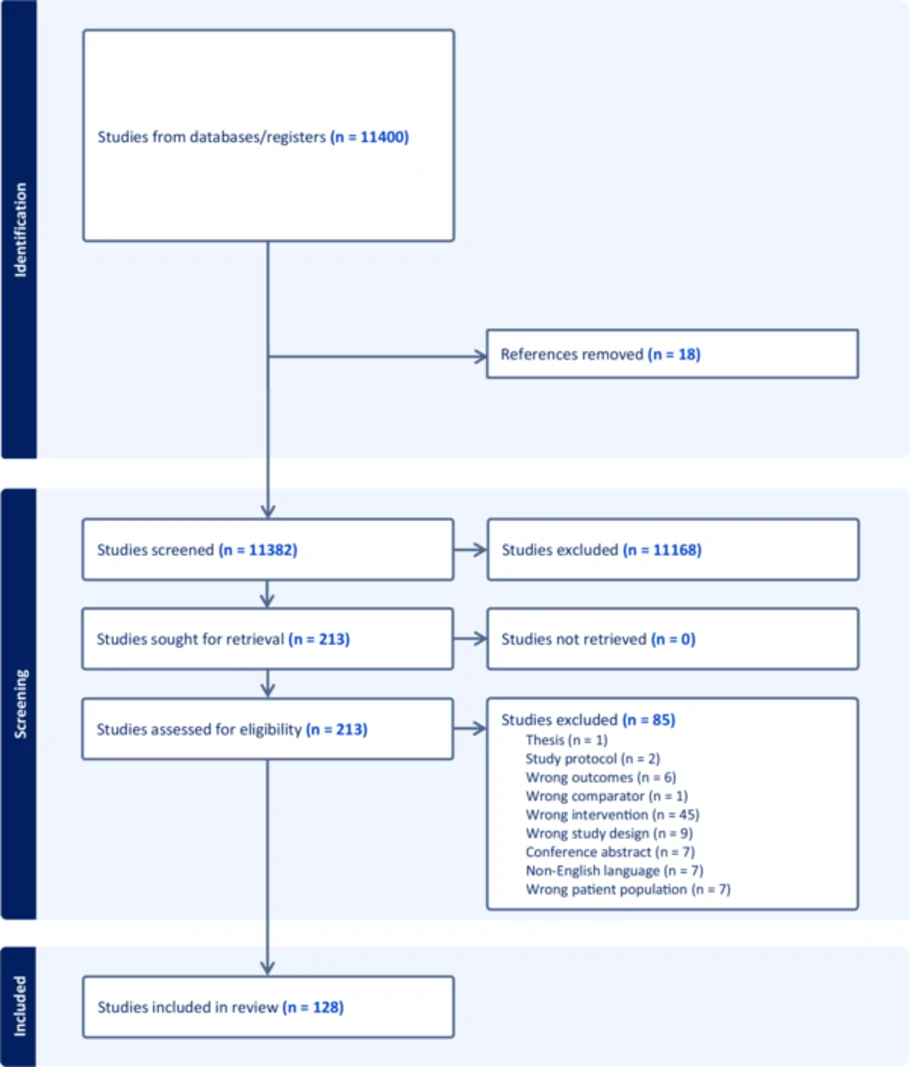
PRISMA flow diagram (taken from Covidence 2020 v1517, Melbourne, Australia).

All screening was completed by six members of the Guideline Steering Group. The titles and abstracts of 11,382 articles were initially screened, from which 213 full‐text articles were independently double‐screened by reviewers. In total, 128 articles (45 RCTs and 83 NRSIs) were included in the review (see Figure 1 for flow of records).

#### 2.4.3 Evidence Synthesis Methods

Data extraction was completed by six members of the Guideline Steering Group, using a self‐developed, customised data extraction template in a Microsoft Excel spreadsheet. All members of the data extraction team completed training on a sample of included studies before commencing formal data extraction, and 20% of data extraction forms were randomly selected to be double‐checked by L.J.

The mean between‐group differences and 95% confidence intervals (95% CI) were extracted from each study if possible. If these data were not available, mean and standard deviation (SD) of change scores provided in the studies or mean (SD) post‐intervention scores were extracted. Where median and interquartile range (IQR) were reported, the mean and SD were calculated [34].

Meta‐analyses were conducted across studies that made similar comparisons if there were at least two studies without excessive clinical heterogeneity. For measures of treatment effect for outcomes measured on the same scale, we calculated the mean difference (MD) and 95% CI using a random‐effects model. Where outcomes were measured using different measures, we calculated the standardised MD (SMD) (Hedges' *g*) and 95% CI using a random‐effects model to pool estimates.

Cochrane RoB tools were used to assess the RoB. For RCTs and cross‐over RCTs, the RoB‐2 [35] was used, while for NRSIs, the ROBINS‐I [36] instrument was used. The level of potential bias was judged as low, high, or unclear for each domain of the relevant tool. Effect sizes were categorised as small (0.1–0.4), medium (0.5–0.7), or large (0.8 or greater). Where it was not possible or appropriate to pool data, study results were narratively synthesised.

#### 2.4.4 Certainty of Evidence Assessment

GRADE‐PRO software [37] was used for creating summary of findings (SoF) tables and populating EtD frameworks. The evidence from the systematic review was independently graded for certainty using the GRADE approach [38] by a single reviewer. The GRADE‐PRO software then provided a GRADE rating for each outcome and comparison. Guideline co‐chair L.J. attended GRADE training (JBI Adelaide GRADE Centre, Nov 2022), while author and methodologist ZM provided GRADE training to all members of the Guideline Steering Group.

#### 2.4.5 Recommendation Grading

The GRADE EtD framework was used to make recommendations for each clinical question. Evidence recommendations were made by initially considering the size and precision of treatment effects along with the certainty of the evidence. The decision as to when to use direct msTBI evidence from RCTs and NRSIs was guided by the process recommended by Cuello‐Garcia et al. [39].

#### 2.4.6 EtD Framework—Other Criteria

Where limited direct evidence was available, data and/or rating of certainty of evidence from other relevant guidelines (i.e., WHO physical activity and sedentary behaviour guideline, stroke (adults), and cerebral palsy (children) guidelines) was added to the EtD frameworks to determine effect and certainty of effect.

The Guideline Steering Group also led three additional studies to inform other aspects of the EtD criteria and future implementation. The studies were (1) a clinical audit of 26 brain injury rehabilitation services across Australia; (2) qualitative research with people with msTBI to understand their experiences of physical activity participation [40]; and (3) qualitative research with six interest‐holder groups to understand barriers and facilitators to physical activity participation. Each study will be reported and published separately. EtD framework uses explicit criteria to generate guideline recommendations [38], which are overviewed in Table 3, including the various sources of evidence contributing to the guideline recommendations. These considerations were documented by three authors (L.H., L.J., B.W.) in EtD tables.

**Table 3:** Types of evidence informing the Evidence to Decision criteria [41].

EtD criteria	Sources of evidence
**Problem** Is the problem a priority?	Direct evidence from msTBI (RCTs, NRSIs) Indirect evidence from general population
**Desirable effects** How substantial are the desirable anticipated effects?	Direct evidence from msTBI (RCTs, NRSIs) Indirect evidence from existing credible guidelines and general population
**Undesirable effects** How substantial are the undesirable anticipated effects?	Direct evidence from msTBI (RCTs, NRSIs) Indirect evidence from existing credible guidelines and general population
**Certainty of evidence** What is the overall certainty of the evidence of effects?	Direct evidence from msTBI (RCTs, NRSIs) Indirect evidence from other clinical conditions (i.e., stroke [adults], CP [children])
**Values** Is there important uncertainty about or variability in how much people value the main outcomes?	Focus groups and interviews with interest‐holders, including people with msTBI
**Balance of effects** Does the balance between desirable and undesirable effects favour the intervention of the comparison?	Based on evidence from preceding criteria
**Resources required** How large are the resource requirements (costs)?	Brain injury rehabilitation services audit Focus groups and interviews with interest‐holders, including people with msTBI
**Certainty of evidence of required resources** What is the certainty of the evidence of resource requirements (costs)?	There is no evidence to guide this judgement in TBI research
**Cost‐effectiveness** Does the cost‐effectiveness of the intervention favour the intervention or the comparison?	There is no evidence to guide this judgement in TBI research
**Equity** What would be the impact on health equity?	Indirect evidence from general population Grey literature including Australian disability and health policies
**Acceptability** Is the intervention acceptable to key interest‐holders?	Direct evidence from msTBI Brain injury rehabilitation services audit Interest‐holder focus groups
**Feasibility** Is the intervention feasible to implement?	Indirect evidence from other clinical conditions (i.e., stroke [adults], CP [children]) Brain injury rehabilitation services audit Focus groups and interviews with interest‐holders, including people with msTBI

Abbreviations: CP, Cerebral Palsy; EtD, Evidence‐to‐Decision; msTBI, moderate to severe traumatic brain injury; NRSIs, Non‐randomised studies of interventions; RCTs, Randomised controlled trials.

### 2.5 Methods for Formulating Recommendations

Following consultation with the Guideline Leadership Group to discuss and plan the Guideline Development Group meetings, the guideline chair (L.H.) and co‐chair (L.J.): (1) approached and appointed an external meeting facilitator with experience in guideline development to run the Guideline Development Group meetings; (2) consulted with author NR, Executive Officer of Brain Injury Australia, the nation's peak body representing Australians with a brain injury, to discuss best practice in including people with lived experience in the Guideline Development Group meetings; (3) disseminated the EtD framework and SoF tables for each clinical question to each member of the Guideline Development Group 2 weeks before meeting; and (4) presented an overview of the guideline development process and what to expect in the Guideline Development Group meetings to people with lived experience online 1‐week before the scheduled meetings (two people with lived experience attended the presentation).

The Guideline Development Group meetings were conducted online (via Zoom) for 13.5 h spread across 5 days over a 3‐week period (July–August 2023) and moderated by a paid facilitator. A standardised process was followed to inform the decision‐making for each recommendation. This included the Guideline Steering Group deciding on a draft rating for each EtD criteria for each question to take to the Guideline Development Group meetings. The Guideline Development Group then either confirmed the draft rating or voted if a change to the EtD criteria judgements were suggested. The direction and strength of each recommendation, and its wording, were independently voted on by the Guideline Development Group members, with 50% agreement required within three rounds of voting for a recommendation, and its wording, to be accepted (Table 4). The meeting attendance and voting records for the EtD criteria judgements (where necessary), direction and strength of recommendations, and the wording of recommendations, can be found in the Guideline Technical Report.

**Table 4:** Summary of evidence for guideline recommendations.

Recommendation	Criteria for justification
For adults and older adults after moderate to severe traumatic brain injury, we suggest regular structured aerobic exercise that is individually‐tailored and across the continuum of care. *Conditional recommendation FOR, low certainty evidence*.	Cardiorespiratory deconditioning is a common problem after msTBI likely to restrict reintegration back into previous roles within family, work and community. Aerobic training is likely to address this problem. *Problem, Desirable Effects, Balance of Effects, Acceptability, Feasibility*.
For children and adolescents after moderate to severe traumatic brain injury, we suggest regular energetic play and/or exercise that is individually tailored and across the continuum of care. *Conditional recommendation FOR, very low certainty evidence*.	Cardiorespiratory deconditioning is a common problem after msTBI likely to restrict reintegration back into previous roles within family, friends, school and community. Aerobic training is likely to address this problem. *Problem, Desirable Effects, Balance of Effects, Acceptability, Feasibility*.
For adults and older adults after moderate to severe traumatic brain injury, we recommend individually‐tailored muscle strengthening exercise, including ballistic training, across the continuum of care. *Strong recommendation FOR, moderate certainty evidence*.	Mobility limitations and muscle weakness are common problems for adults and older adults after msTBI. Ballistic exercise training can improve mobility, while muscle strengthening can improve muscle weakness. *Problem, Desirable Effects, Certainty of Evidence, Balance of Effects, Acceptability, Feasibility*.
For children and adolescents after moderate to severe traumatic brain injury, we suggest regular muscle strengthening play and/or exercise that is individually‐tailored and across the continuum of care. *Conditional recommendation FOR*.*	Muscle strength is impaired after msTBI, which can restrict participation in meaningful activities such as sport and play. The WHO recommend muscle strength training for children and adolescents living with a disability. *Problem, Desirable Effects, Undesirable Effects, Acceptability, Feasibility*.
For adults and older adults after moderate to severe traumatic brain injury, we recommend task‐specific mobility training across the continuum of care. *Strong recommendation FOR, moderate certainty evidence*.	Reduced mobility is a common problem after msTBI with negative consequences. Indirect evidence from stroke rehabilitation and motor learning principles strongly supports mobility training. *Problem, Desirable Effects, Balance of Effects, Acceptability, Feasibility*.
For children and adolescents after moderate to severe traumatic brain injury, we suggest task‐specific mobility training across the continuum of care. *Conditional recommendation FOR, very low certainty evidence*.	Reduced mobility is a common problem after msTBI with negative consequences. Mobility training is likely to address this problem. *Problem, Desirable Effects, Balance of Effects, Acceptability, Feasibility*.
For adults and older adults after moderate to severe traumatic brain injury, we suggest participation in sport^a^ and physical recreation^b^ across the continuum of care considering their personal preference and capability. *Conditional recommendation FOR, very low certainty evidence*.	Physical inactivity is highly prevalent and problematic for adults and older adults after msTBI. Sport and physical recreation programs can provide opportunities to be physically active in a safe, social, and supportive environment. *Problem, Balance of Effects, Resources Required, Acceptability, Feasibility*.
For children and adolescents after moderate to severe traumatic brain injury, we suggest participation in sport^a^ and physical recreation^b^ across the continuum of care considering their personal preference and capability. *Conditional recommendation FOR*.*	Physical inactivity is highly prevalent and problematic for children and adolescents after msTBI. Sport and physical recreation programs can provide opportunities for children and adolescents to engage in physical activity in a social and supportive environment. *Problem, Desirable Effects, Balance of Effects, Acceptability, Feasibility*.
For adults and older adults after moderate to severe traumatic brain injury, we suggest the promotion of physical activity across the continuum of care. *Conditional recommendation FOR, low certainty evidence*.	Adults and older adults after msTBI are typically physically inactive. The promotion of overall physical activity to adults and older adults after msTBI may increase their physical activity levels and participation. *Problem, Certainty of Evidence, Balance of Effects, Equity, Feasibility*.
For children and adolescents after moderate to severe traumatic brain injury, we suggest the promotion of physical activity across the continuum of care. *Conditional recommendation FOR*.*	Physical inactivity can lead to an array of negative health consequences for children and adolescents. Overall physical activity promotion can encourage children and adolescents after msTBI to be physically active. *Problem, Desirable Effects, Balance of Effects, Acceptability, Feasibility*.

*Note:* For further information about the Guideline recommendations, including summary of findings and EtD tables, forest plots, risk of bias assessments, etc., please refer to the Guideline Technical Report. Unshaded rows are for adults and older adults and the shaded rows are for children and adolescents.

Abbreviations: msTBI, moderate to severe traumatic brain injury; WHO, World Health Organization.

^a^ ‘An activity involving physical exertion, skill and/or hand‐eye coordination as the primary focus of the activity, with elements of competition where rules and patterns of behaviour governing the activity exist formally through organisations’ [42].

^b^ ‘An activity or experience that involves varying levels of physical exertion, prowess and/or skill, which may not be the main focus of the activity and is voluntarily engaged in by an individual in leisure time for the purpose of mental and/or physical satisfaction’ [42].

* In the absence of direct evidence, certainty of evidence could not be assessed.

The language used to describe the direction (i.e., ‘FOR’ or ‘AGAINST’) and strength (i.e., ‘Strong’ or ‘Conditional’) of each recommendation was based on the GRADE approach [10]. Definitions from the GRADE Handbook were used throughout the guideline development process [38].

#### 2.5.1 Development of Good Practice Points

Based on the expert opinion and lived experience of the Guideline Development Group members, and evidence provided from the included studies, good practice points and precautionary practice points were developed to further guide clinical decision‐making alongside the guideline recommendations.

## 3 External Review and Guideline Update

### 3.1 External Review

The guideline underwent independent review as per the Australian National Health and Medical Research Council (NHMRC) ‘Guidelines for Guidelines’ processes (NHMRC, 2023). Six individuals with expertise in TBI, disability, and/or physical activity, were nominated by the guideline chair and co‐chair, and the NHMRC invited reviewers from this list to independently review the draft guideline. The NHMRC also commissioned an external methodological review of the guideline. The guideline was made available for public consultation via the Connectivity TBI website between 4/9/2023 and 6/10/2023, accompanied by a REDCap (Research Electronic Data Capture) survey used to gather public feedback. Invitations for public consultation were also sent directly to relevant peak representative bodies (i.e., Australian Physiotherapy Association [APA], Exercise and Sports Science Australia [ESSA], and the Rehabilitation Medicine Society of Australia and New Zealand [RMSANZ]), 10 Australian state and commonwealth authorities, and two patient advocacy groups (i.e., Brain Injury Australia, Heads Together for ABI). A link to the draft guideline and feedback form was also circulated in the fortnightly ‘NHMRC Tracker’ newsletter (11/09/2023), via the personal social media accounts of the guideline chair and co‐chair, and institutional‐linked social media accounts. Based on these reviews, a total of 50 comments were addressed and incorporated into the guideline. The outcome of the public consultation can be accessed through the public consultation report.

The updated guideline underwent AGREE II [43] assessments conducted independently by two reviewers invited by the chair and co‐chair (L.H. and L.J., respectively). The guideline was then formally submitted to the NHMRC Guideline council on 1 February, 2024, undergoing further review before a council meeting attended by L.H. and L.J. on 28 March, 2024. The guideline was approved by the Chief Executive Officer of the NHMRC on the advice of the NHMRC Guideline council on 18 April, 2024, and endorsed by the APA, ESSA, and RMSANZ. The guideline was launched online on 27 August, 2024, and is freely accessible at https://www.imh.org.au/bridges.

### 3.2 Plans for Updating This Guideline

The BRIDGES Guideline Development Group recommends that the guideline be reviewed, assessed for the need to be updated, and new or modified recommendations developed, within 5 years of publication, or earlier if significant new research emerges warranting change.

### 3.3 Updating or Adapting Recommendations Locally

The guideline has been informed by research studies conducted globally and contextualised to Australian settings and people by the BRIDGES brain injury rehabilitation services audit, and qualitative research with interest‐holder groups. The BRIDGES group is currently working with brain injury rehabilitation services to implement the guideline recommendations for their service. This includes the development of local resources (e.g., policies and procedures, funding templates) and conducting a hybrid type III implementation‐effectiveness RCT across Australia, funded by the Medical Research Future Fund.

## 4 Implementation Considerations

Implementation science methods [44] were used to guide the development of this clinical practice guideline to optimise its suitability for future implementation [20]. This included using frameworks and models to identify likely determinants of implementation [45]. Barriers (and some facilitators) to implementation have been identified in the outer context (e.g., funding legislature and complexity), in the inner context where the guideline will be implemented (i.e., brain injury rehabilitation services) (e.g., resources), for the people delivering the guideline recommended physical activity (i.e., health professionals such as physiotherapists and exercise physiologists) (e.g., knowledge and skills) and for the people receiving the guideline recommended physical activity (i.e., people with msTBI) (e.g., fatigue) [46]. Implementation strategies to address the barriers and facilitators identified will be selected and tested in the hybrid type III RCT to implement the guideline into practice across Australia. Additional work is also underway in priority populations with msTBI where additional barriers (e.g., language, cultural competence, access) to participating in physical activity and interacting with health services are likely. These include Aboriginal and Torres Strait Islander people, people from Culturally and Linguistically Diverse communities, people living in rural and remote Australia and people with high support needs.

## 5 Discussion

Using the GRADE ADOLOPMENT [22] methodology for guideline development, we have developed the first Australian Physical Activity Clinical Practice Guideline for people with msTBI. The guideline incorporates 10 *de novo* recommendations to support health professionals' clinical decision‐making and increase uptake of physical activity by people with msTBI. There is limited direct evidence for the prescription and promotion of physical activity for people with msTBI. To counter this lack of direct evidence, we used a rigorous and systematic approach, and involved key interest‐holders, including people with msTBI and those responsible for the promotion and prescription of physical activity (i.e., physiotherapists, exercise physiologists), to formulate the guideline.

The conception of the Australian Physical Activity Clinical Practice Guideline for people with msTBI was based on the premise of ‘adapting’ or ‘adopting’ the 2020 WHO physical activity guidelines for people with disability to people with msTBI across the continuum of rehabilitation care. The final guideline, though, consists of a series of *de novo* recommendations established on a broad body of msTBI and non‐TBI (i.e., stroke, cerebral palsy) research evidence, a clinical audit of 26 brain injury services in Australia, and interest‐holder consultations and focus groups, including people with msTBI. The recommendations made are still consistent with the recommendations in the WHO physical activity guideline for people with disability [17]. Expanding on the WHO recommendations, they are suitable for rehabilitation within the continuum of care post‐injury. The evidence of risks associated with physical activity for people with msTBI are minimal, and while direct evidence for the benefits of physical activity continues to emerge, evidence from non‐TBI populations would suggest the benefits certainly outweigh the potential harms for people with msTBI.

The prescription of individually‐tailored physical activity interventions should be standard clinical practice for people with msTBI in Australian rehabilitation settings, while accessible opportunities to be active in the community should be facilitated. While further research is needed to support the implementation of the guideline into clinical practice, pre‐implementation work conducted as part of the development of the guideline suggests implementation of the recommendations is both feasible and likely to be acceptable. However, variations in current practice and barriers to implementation have been identified as part of guideline development and will need resolving for people with msTBI to benefit from this study.

## 6 Research Priorities

With the limited high‐quality direct evidence to guide clinical practice for the prescription and promotion of physical activity, there is a need for rigorous studies across the physical activity interventions included in this guideline. Areas where evidence was particularly limited or non‐existent included children and adolescents, older adults, and inpatient rehabilitation in people < 1‐year post‐TBI event (see Table 4). Deciding on priority questions should be conducted with key interest‐holders, in particular people with msTBI, to ensure that the most important questions are addressed first. Collaborations between consumer organisations, academics, and clinical services and creating learning healthcare systems where data can be collected and used for clinical and research purposes will also assist.

No data were identified informing resource requirements (costings) for delivering and promoting physical activity in health systems. Costing analysis studies to inform decisions around costs of physical activity participation and cost‐effectiveness of physical activity delivery in brain injury health services are warranted.

## 7 Other Guidelines

As part of the GRADE‐ADOLOPMENT process *identification of credible existing guidelines*, we searched online databases for national and international guidelines relevant to the promotion and prescription of physical activity by health professionals to people with msTBI. Content experts (i.e., msTBI, physical activity) were also consulted to identify any relevant guidelines that may not have been captured by the search. While no guidelines were selected as being appropriate for adaptation or adoption, the Evidence‐based Review of moderate to severe Acquired Brain Injury (ERABI) [47] and International Cognitive (INCOG) 2.0 Guidelines for Cognitive Rehabilitation Following Traumatic Brain Injury [48] were identified as having some relevance (i.e., scope and purpose, target users, target population) to this guideline.

- The ERABI suggests gait and mobility training, virtual reality‐based therapy, and yoga can improve motor function. While aerobic exercise programs can be beneficial to motor function, balance and cardiovascular parameters [47].
- The INCOG 2.0 Guidelines recommend physical therapy for patients in posttraumatic amnesia, with dose and location of rehabilitation based on the patient's fatigue, degree of agitation, and cognitive impairment [48].

The recommendations are consistent with the recommendations from this guideline despite the methodological differences between the guidelines.

## 8 Conclusion

People with msTBI are highly inactive and are often deprived of the health and social benefits of physical activity, putting them at risk of chronic health conditions. The Australian Physical Activity Clinical Practice Guideline for people with msTBI was developed using a GRADE ADOLOPMENT methodology and informed by both direct TBI evidence and indirect evidence from relevant health conditions. The Guideline features 10 *de novo* recommendations to support health professionals to provide and promote physical activity to people with msTBI.

## Author Contributions

**Leanne Hassett:** conceptualisation, formal analysis, funding acquisition, investigation, methodology, project administration, supervision; writing – original draft, writing – review and editing. **Liam Johnson:** formal analysis, investigation, methodology, project administration, supervision, validation, writing – original draft, writing – review and editing. **Gavin Williams:** conceptualisation, funding acquisition, investigation, methodology, supervision, writing–review and editing. **Rhys Ashpole:** funding acquisition, investigation, writing – review and editing. **Adrian Bauman:** conceptualisation, funding acquisition, investigation, writing – review and editing. **Catherine Carty:** investigation, methodology, supervision, writing – review and editing. **Sakina Chagpar:** formal analysis, investigation, project administration, writing – review and editing. **Kelly Clanchy:** funding acquisition, investigation, writing – review and editing. **Abby Haynes:** funding acquisition, investigation, methodology, project administration, supervision, writing – review and editing. **Kate Heine:** investigation, writing – review and editing. **Zachary Munn:** investigation, methodology, supervision, writing – review and editing. **Anthony Okely:** investigation, methodology, supervision, writing – review and editing. **Nick Rushworth:** funding acquisition, investigation, writing – review and editing. **Adam Scheinberg:** funding acquisition, investigation, writing – review and editing. **Catherine Sherrington:** conceptualisation, funding acquisition, investigation, methodology, writing – review and editing. **Grahame Simpson:** funding acquisition, investigation, writing – review and editing. **Anne Tiedemann:** fnding acquisition, investigation, writing – review and editing. **Sean Tweedy:** funding acquisition, investigation, writing – review and editing. **Gabrielle Vassallo:** funding acquisition, investigation, writing – review and editing. **Belinda Wang:** formal analysis, investigation, validation, writing – review and editing. **Kerry West:** investigation, writing – review and editing. **Luke Wolfenden:** conceptualisation, funding acquisition, investigation, writing – review and editing.

## BRIDGES Guideline Development Group Members

*Pien Alferink*, Institute for Musculoskeletal Health, Sydney Local Health District, Gadigal Country, Sydney, Australia; *Rhys Ashpole*, icare NSW, Sydney, Australia; *Adrian Bauman*, Sydney School of Public Health, Faculty of Medicine and Health, The University of Sydney, Sydney, Australia; *Olivia Beattie*, The Royal Children's Hospital, Melbourne, Australia; *Francesca Brady*, Person with Lived Experience; *Catherine Carty*, Munster Technological University, UNESCO Chair Manager, Kerry, Ireland; *Sakina Chagpar*, Institute for Musculoskeletal Health, Sydney Local Health District, Gadigal Country, Sydney, Australia; *Daniel Cheung*, Institute for Musculoskeletal Health, Sydney Local Health District, Gadigal Country, Sydney, Australia; Sydney School of Health Sciences, Faculty of Medicine and Health, The University of Sydney, Sydney, Australia; *Kelly Clanchy*, School of Health Sciences and Social Work, Griffith Health, Griffith University, Gold Coast, Australia; The Hopkins Centre, Griffith University, Nathan, Australia; *Alexandra Edmondson*, Institute for Musculoskeletal Health, Sydney Local Health District, Gadigal Country, Sydney, Australia; *Joanne Glinsky*, Sydney School of Health Sciences, Faculty of Medicine and Health, The University of Sydney, Sydney, Australia; John Walsh Centre for Rehabilitation Research, Kolling Institute, Northern Sydney Local Health District and The University of Sydney, Sydney, Australia; *Leanne Hassett*, Institute for Musculoskeletal Health, Sydney Local Health District, Gadigal Country, Sydney, Australia; Sydney School of Health Sciences, Faculty of Medicine and Health, The University of Sydney, Sydney, Australia; *Abby Haynes*, Institute for Musculoskeletal Health, Sydney Local Health District, Gadigal Country, Sydney, Australia; Sydney School of Public Health, Faculty of Medicine and Health, The University of Sydney, Sydney, Australia; *Kate Heine*, Heads Together for Brain Injury, Melbourne, Australia; *Sonia Hoppe*, School of Health and Rehabilitation Sciences, The University of Queensland, Brisbane, Australia; *Liam Johnson*, Institute for Musculoskeletal Health, Sydney Local Health District, Gadigal Country, Sydney, Australia; School of Health Sciences, Faculty of Medicine, Dentistry and Health Sciences, University of Melbourne, Melbourne, Australia; Physiotherapy Department, Epworth HealthCare, Melbourne, Australia; School of Behavioural and Health Sciences, Faculty of Health Sciences, Australian Catholic University, Melbourne, Australia; *Anthony Mamo*, Person with Lived Experience, Sydney, Australia; *Peter Mayhew*, Person with Lived Experience, Perth, Australia; *Zachary Munn*, Health Evidence Synthesis, Recommendations and Impact (HESRI), School of Public Health, Faculty of Health and Medical Sciences, University of Adelaide, Adelaide, South Australia, Australia; *Anthony Okely*, School of Health and Society, University of Wollongong, Wollongong, Australia; *Nick Rushworth*, Brain Injury Australia, Sydney, Australia; *Sania Salim*, The Royal Children's Hospital, Melbourne, Australia; *Benjamin Sammut*, Person with Lived Experience; *Adam Scheinberg*, Murdoch Children's Research Institute, Melbourne, Australia; *Catherine Sherrington*, Institute for Musculoskeletal Health, Sydney Local Health District, Gadigal Country, Sydney, Australia; Sydney School of Public Health, Faculty of Medicine and Health, The University of Sydney, Sydney, Australia; *Grahame Simpson*, Sydney School of Health Sciences, Faculty of Medicine and Health, The University of Sydney, Sydney, Australia; Liverpool Brain Injury Rehabilitation Unit, South Western Sydney Local Health District, Sydney, Australia; John Walsh Centre for Rehabilitation Research, Kolling Institute, Northern Sydney Local Health District and The University of Sydney, Sydney, Australia; *Anne Tiedemann*, Institute for Musculoskeletal Health, Sydney Local Health District, Gadigal Country, Sydney, Australia; Sydney School of Public Health, Faculty of Medicine and Health, The University of Sydney, Sydney, Australia; *Sean Tweedy*, School of Human Movement and Nutrition Sciences, Faculty of Health and Behavioural Sciences, University of Queensland, Brisbane, Australia; Health and Wellbeing Centre for Research Innovation, The University of Queensland, St Lucia, Australia; Queensland Centre for Olympic and Paralympic Studies, The University of Queensland, St Lucia, Australia; *Gabrielle Vassallo*, Consumer representative, Sydney, Australia; *Sarah Veli‐Gold*, Sydney School of Health Sciences, Faculty of Medicine and Health, The University of Sydney, Sydney, Australia; *Belinda Wang*, Institute for Musculoskeletal Health, Sydney Local Health District, Gadigal Country, Sydney, Australia; Sydney School of Public Health, Faculty of Medicine and Health, The University of Sydney, Sydney, Australia; *Nicholas Waters*, Person with Lived Experience, Melbourne, Australia; *Kerry West*, Institute for Musculoskeletal Health, Sydney Local Health District, Gadigal Country, Sydney, Australia; Sydney School of Public Health, Faculty of Medicine and Health, The University of Sydney, Sydney, Australia; Physiotherapy Department, The Children's Hospital at Westmead, Sydney, Australia; *Bhavini Whiteside*, Liverpool Brain Injury Rehabilitation Unit, South Western Sydney Local Health District, Sydney, Australia; *Gavin Williams*, School of Health Sciences, Faculty of Medicine, Dentistry and Health Sciences, University of Melbourne, Melbourne, Australia; Physiotherapy Department, Epworth HealthCare, Melbourne, Australia; *Luke Wolfenden*, Hunter New England Local Health District, University of Newcastle, Newcastle, Australia.

## National Health and Medical Research Council Approval

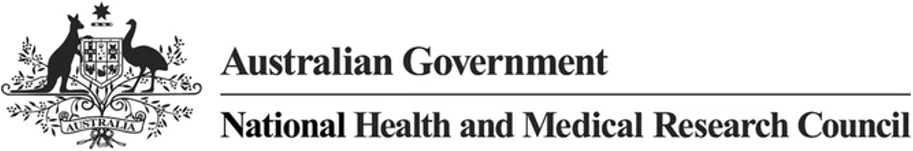

The guideline recommendations on pages 4, and 9–10 of this document were approved by the Chief Executive Officer of the National Health and Medical Research Council [49] on 18 April 2024 under section 14A of the *National Health and Medical Research Council Act 1992*. In approving the guideline recommendations, NHMRC considers that they meet the NHMRC standard for clinical practice guidelines. This approval is valid for a period of 5 years.

NHMRC is satisfied that the guideline recommendations are systematically derived, based on the identification and synthesis of the best available scientific evidence, and developed for health professionals practising in an Australian health care setting.

This publication reflects the views of the authors and not necessarily the views of the Australian Government.

## Conflicts of Interest

The guideline was produced in accordance with the processes outlined in the BRIDGES Physical Activity Clinical Practice Guideline for people with moderate to severe TBI conflict of interest (CoI) policy (Appendix S1). CoI disclosures are available in Appendix S2.

## Supporting information


**Appendix 1:** CoI policy.

**Appendix 2:** CoI Disclosures.

**Appendix 3:** Guideline Groups.

**Appendix 4:** Critical and Important outcomes.

**Appendix 5:** Identification of credible existing guidelines and AGREE ratings.

**Appendix 6:** Stroke systematic reviews.

**Appendix 7:** Systematic review methods and evidence synthesis.

**Appendix 8:** Systematic review search strategy.

## Data Availability

Relevant extracted data used in this guideline have been reported in the text, figures, and tables (including Appendices). All other extracted data used in the guideline are available in the reports located on the Institute for Musculoskeletal Health, University of Sydney, https://www.imh.org.au/bridges.
