# Supplementary material for: A Physical Activity Clinical Practice Guideline for People With Moderate to Severe Traumatic Brain Injury
Source: Clin Public Health Guidel. 2025 Aug 14;2(4):e70035. doi: 10.1002/gin2.70035 (PMC13563333; doi:10.1002/gin2.70035)
Supplement: Supplementary file 1 [file GIN2-2-e70035-s001.docx]

**Identification of credible existing guidelines**

| **CRITERIA** | **GUIDELINES** | | | | | | | | | | | | |
| --- | --- | --- | --- | --- | --- | --- | --- | --- | --- | --- | --- | --- | --- |
|  | ***WHO Guidelines^1^*** | ***UK PA Guidelines^2^*** | ***SIGN Guidelines^3^*** | ***Americans PA Guidelines^4^*** | ***ONF Guidelines^5^*** | ***SCI Guidelines^6^*** | ***BSRM Guidelines^7^*** | ***SCI Position Stand^8^*** | ***MS Guidelines^9^*** | ***NZ Guidelines^10^*** | ***Stroke Guidelines^11^*** | ***CP Motor Rehab Guidelines^12^*** | ***Children and Young People CP Guidelines^13^*** |
| **Published in last 10 years** | Yes | Yes | Yes | Yes | Yes | Yes | No | Yes | Yes | No | Yes | Yes | Yes |
| **Followed GRADE Process** | Yes | Yes | No | No | No | Yes | No | No | No | No | Yes | Unsure | Yes |
| **Addresses clear questions (can identify PICO elements)** | Yes | No | Yes | Yes | No | Yes | No | No | Yes | Yes | Yes | Yes | Yes |
| **Has benefits and harms assessments for patient-important outcomes** | Yes | Yes | Yes | Yes | No | Yes | Yes | Yes | Yes | Yes | Yes | Yes | Yes |
| **Assessed using AGREE** | No | No | No | No | No | Yes | No | No | Yes | No | No | Yes | No |
| **Allows for updating (e.g., present full systematic reviews, accessible search strategy, analysis method)** | Yes | No | No | Yes | No | Yes | No | No | Yes | Yes | Yes | Yes | Yes |
| **Has existing and accessible evidence-tables/summaries** | Yes | No | No | No | No | Yes | No | No | No | Yes | Yes | Unsure | Yes |
| **Has risk of bias assessment** | Yes | No | No | Yes | No | Yes | No | No | No | No | Yes | No | Yes |
| **Costs associated with implementing guideline** | Yes | No | Yes | No | No | Yes | No | No | Yes | No | Yes | No | Yes |
| **Accompany - how they are going to implement - disseminate the guidelines** | Yes | Yes | Yes | Yes | No | Yes | No | No | Yes | Yes | Yes | Yes | Yes |

WHO, World Health Organisation; UK, United Kingdom; PA, Physical Activity; SIGN, Scottish Intercollegiate Guidelines Network; ONF, Ontario Neurotrauma Foundation; SCI, Spinal Cord Injury; BSRM, British Society of Rehabilitation Medicine; MS, Multiple Sclerosis; NZ, New Zealand; CP, Cerebral Palsy; GRADE, Grading of Recommendations Assessment, Development and Evaluation; PICO, Population, Intervention, Comparison, Outcome; AGREE, Appraisal of Guidelines, Research and Evaluation.

**AGREE-II Ratings**

| **AGREE-II^14^** | | **Children and Young People CP Guidelines^13^** | | **WHO Guidelines^1^** | | **Stroke Guidelines^11^** | |
| --- | --- | --- | --- | --- | --- | --- | --- |
| **Section** | **Item** | **Appraiser 2** | **Appraiser 1** | **Appraiser 2** | **Appraiser 1** | **Appraiser 1** | **Appraiser 2** |
| Scope and Purpose | 1 | 7 | 7 | 7 | 7 | 7 | 7 |
| Scope and Purpose | 2 | 7 | 7 | 7 | 7 | 7 | 7 |
| Scope and Purpose | 3 | 7 | 7 | 7 | 7 | 7 | 7 |
| Stakeholder Involvement | 4 | 6 | 7 | 7 | 7 | 7 | 7 |
| Stakeholder Involvement | 5 | 7 | 7 | 6 | 7 | 7 | 7 |
| Stakeholder Involvement | 6 | 7 | 7 | 7 | 7 | 7 | 7 |
| Rigour of Development | 7 | 7 | 7 | 6 | 6 | 7 | 5 |
| Rigour of Development | 8 | 7 | 7 | 7 | 7 | 6 | 6 |
| Rigour of Development | 9 | 7 | 7 | 7 | 7 | 7 | 7 |
| Rigour of Development | 10 | 7 | 7 | 7 | 7 | 6 | 7 |
| Rigour of Development | 11 | 7 | 7 | 7 | 7 | 7 | 7 |
| Rigour of Development | 12 | 7 | 7 | 7 | 7 | 7 | 7 |
| Rigour of Development | 13 | 7 | 6 | 6 | 7 | 7 | 4 |
| Rigour of Development | 14 | 4 | 6 | 6 | 6 | 7 | 7 |
| Clarity of Presentation | 15 | 7 | 7 | 7 | 7 | 6 | 6 |
| Clarity of Presentation | 16 | 7 | 6 | 7 | 6 | 6 | 7 |
| Clarity of Presentation | 17 | 7 | 7 | 7 | 7 | 5 | 6 |
| Applicability | 18 | 6 | 7 | 4 | 7 | 6 | 7 |
| Applicability | 19 | 7 | 7 | 5 | 7 | 6 | 7 |
| Applicability | 20 | 7 | 7 | 7 | 7 | 6 | 6 |
| Applicability | 21 | 2 | 5 | 5 | 7 | 7 | 7 |
| Editorial Independence | 22 | 7 | 7 | 5 | 6 | 6 | 7 |
| Editorial Independence | 23 | 7 | 7 | 7 | 7 | 6 | 7 |
| Overall Assessment | OA1 | 6 | 7 | 7 | 7 | 6 | 6 |
| Overall Assessment | OA2 | Yes | Yes with modifications | Yes | Yes | Yes with modifications | Yes with modifications |

AGREE, Appraisal of Guidelines, Research and Evaluation; CP, Cerebral Palsy; WHO, World Health Organisation; OA, Overall Assessment.

^1^ WHO guidelines on physical activity and sedentary behaviour. Geneva: World Health Organization; 2020. (Accessed on 8 Feb 2022). Available online: <https://www.who.int/publications/i/item/9789240015128>

^2^ Chief Medical Officer’s Physical Activity Guidelines. 2019. (Accessed on 9 Nov 2022). Available online: <https://assets.publishing.service.gov.uk/government/uploads/system/uploads/attachment_data/file/832868/uk-chief-medical-officers-physical-activity-guidelines.pdf>

^3^ Scottish Intercollegiate Guidelines Network (SIGN). Brain injury rehabilitation in adults. Edinburgh: SIGN; 2013. [Accessed 4 Nov 2022]. Available online: <https://www.sign.ac.uk/media/1068/sign130.pdf>

^4^ U.S. Department of Health and Human Services. Physical Activity Guidelines for Americans, 2nd edition. Washington, DC: U.S. Department of Health and Human Services; 2018. [Accessed 10 Nov 2022]. Available online: <https://health.gov/sites/default/files/2019-09/Physical_Activity_Guidelines_2nd_edition.pdf>

^5^ Lamontagne M-E, Truchon C, Kagan C, et al. INESSS-ONF Clinical practice guidelines for the rehabilitation of adults having sustained a moderate-to-severe TBI. 2017. [Accessed 11 Nov 2022]. Available online: <https://kite-uhn.com/brain-injury/en>

^6^ Martin-Ginis KA, Hicks AL, Latimer AE et al. The development of evidence-informed physical activity guidelines for adults with spinal cord injury. *Spinal Cord*. 2011; 49(11): 1088-1096.

^7^ Royal College of Physicians and British Society of Rehabilitation Medicine. Rehabilitation following acquired brain injury: national clinical guidelines (Turner-Stokes L, ed). London: RCP, BSRM, 2003. [Accessed 15 Nov 2022]. Available online: <https://www.headway.org.uk/media/3320/bsrm-rehabilitation-following-acquired-brain-injury.pdf>

^8^ Tweedy SM, Beckman EM, Geraghty TJ, et al. Exercise and sports science Australia (ESSA) position statement on exercise and spinal cord injury. *J Sci Med Sport.* 2017; 20(2): 108-115.

^9^ Latimer-Cheung AE, Martin Ginis KA, Hicks AL, et al. Development of evidence-informed physical activity guidelines for adults with multiple sclerosis. *Arch Phys Med Rehabil.* 2013; 94(9): 1829-1836.

^10^ Traumatic Brain Injury: Diagnosis, Acute Management and Rehabilitation [Internet]. 1st ed Wellington: New Zealand Guidelines Group; 2006. [Accessed 18 Nov 2022]. Available online: <https://www.moh.govt.nz/notebook/nbbooks.nsf/0/B8738C3605889A6ACC257A6D00809243/$file/traumatic-brain-injury-acc.pdf>

^11^ Stroke Foundation. Clinical Guidelines for Stroke Management. Available at https://informme.org.au/guidelines/living-clinical-guidelines-for-stroke-management. [Accessed 16 Nov 2022]. Available online: <https://app.magicapp.org/#/guideline/Kj2R8j>

^12^ Verschuren O, Peterson MD, Balemans ACJ, Hurvitz EA. Exercise and physical activity recommendations for people with cerebral palsy. *Dev Med Child Neurol*. 2016; 58(8): 798-808.

^13^ Jackman M, Sakzewski L, Morgan C, et al. Interventions to improve physical function for children and young people with cerebral palsy: international clinical practice guideline. *Dev Med Child Neurol*. 2022; 64(5): 536-549.

^14^ AGREE Next Steps Consortium. The AGREE II Instrument [Electronic version]. 2013. [Accessed 27 Nov 2022]. Available online: <http://www.agreetrust.org>
