# Supplementary material for: A Physical Activity Clinical Practice Guideline for People With Moderate to Severe Traumatic Brain Injury
Source: Clin Public Health Guidel. 2025 Aug 14;2(4):e70035. doi: 10.1002/gin2.70035 (PMC13563333; doi:10.1002/gin2.70035)
Supplement: Supplementary file 2 [file GIN2-2-e70035-s002.docx]

**Guideline Steering Group**

A Guideline Steering Group was convened to carry out the systematic review, including the search strategy, title, abstract and full-text screening, and data extraction; administrative tasks associated with organising and carrying out the Guideline Development Group meetings; collecting and interpreting data indirectly informing the guidelines (i.e., BRIDGES [BRain Injury: Developing GuidElineS for physical activities] brain injury services clinical audit, stakeholder focus groups, one-on-one interviews and focus groups with people with lived experience). The group was assembled by the chief investigator.

**Guideline Steering Group members**

| **Name (role)** | **Affiliation** |
| --- | --- |
| A/Prof Leanne Hassett (BRIDGES Chief Investigator and Guideline Chair) | - Institute for Musculoskeletal Health, Sydney Local Health District, Gadigal Country, Sydney, Australia. - Sydney School of Health Sciences, Faculty of Medicine and Health, The University of Sydney, Sydney, Australia. |
| Dr Liam Johnson (BRIDGES Postdoctoral Research Fellow and Guideline Co-chair) | - Institute for Musculoskeletal Health, Sydney Local Health District, Gadigal Country, Sydney, Australia. - School of Health Sciences, Faculty of Medicine, Dentistry and Health Sciences, University of Melbourne, Melbourne, Australia. - Physiotherapy Department, Epworth HealthCare, Melbourne, Australia. - School of Behavioural and Health Sciences, Faculty of Health Sciences, Australian Catholic University, Melbourne, Australia. |
| Dr Abby Haynes (Oversee BRIDGES qualitative research) | - Institute for Musculoskeletal Health, Sydney Local Health District, Gadigal Country, Sydney, Australia. - Sydney School of Public Health, Faculty of Medicine and Health, The University of Sydney, Sydney, Australia. |
| Ms Sakina Chagpar (BRIDGES Research Officer) | - Institute for Musculoskeletal Health, Sydney Local Health District, Gadigal Country, Sydney, Australia. |
| Ms Belinda Wang (BRIDGES Research Assistant) | - Institute for Musculoskeletal Health, Sydney Local Health District, Gadigal Country, Sydney, Australia. - Sydney School of Public Health, Faculty of Medicine and Health, The University of Sydney, Sydney, Australia. |
| Ms Kerry West (BRIDGES Research Officer) | - Institute for Musculoskeletal Health, Sydney Local Health District, Gadigal Country, Sydney, Australia. - Sydney School of Public Health, Faculty of Medicine and Health, The University of Sydney, Sydney, Australia. - Physiotherapy Department, The Children’s Hospital at Westmead, Sydney, Australia. |
| Ms Pien Alferink (BRIDGES Research Student) | - Institute for Musculoskeletal Health, Sydney Local Health District, Gadigal Country, Sydney, Australia. |
| Ms Ella Bracone (BRIDGES Research Student) | - Sydney School of Public Health, Faculty of Medicine and Health, The University of Sydney, Sydney, Australia. |

BRIDGES, BRain Injury: Developing GuidElineS for physical activities

**Guideline Leadership Group**

A Guideline Leadership Group was convened to oversee the development of the Australian Physical Activity Clinical Practice Guideline for people with moderate to severe traumatic brain injury (TBI). The group was assembled by the chief investigator. The purpose of the Guideline Leadership Group was oversight and governance of the project.

*In scope:*

- Oversee the process, management, governance and administration of the guideline.
- Provide recommendations about the process, management, governance and administration of the guideline.

*Out of scope:*

- Decisions about the Evidence-to-Decision (EtD) framework criteria judgements and recommendations, and approval of the recommendations, within the clinical practice guideline for health professionals. This was the responsibility of the Guideline Development Group.

*Membership:*

Membership of the Guideline Leadership Group included the project chief investigator and postdoctoral research fellow (chair and co-chair), a member with lived experience of TBI and content experts (guideline methodology, TBI physiotherapy and exercise physiology, TBI research/evidence, physical activity interventions and guidelines, paediatric rehabilitation, adult rehabilitation). Key responsibilities of the Guideline Leadership Group included (adapted from National Institute for Health and Care Excellence [NICE], 2014)

*All members:*

- Contributed to meetings and declared relevant conflicts of interest.
- Decisions about the clinical questions covered in the clinical guideline.
- Contributed and provided strategies for resolution of issues within the project as/if they occurred.
- Considered and contributed to suggestions about guideline reviewers and Guideline Development Group membership.
- Contributed to ranking of outcomes.
- Attended Grading of Recommendations Assessment, Development and Evaluation (GRADE) training workshop led by group member methodologist.

*Chair and co-chair:*

- Facilitated participation of Guideline Leadership Group members.
- Managed conflicts of interest.
- Updated the Guideline Leadership Group on project milestones and developments between meetings.
- Organised administration and receipt of tasks as required of the Guideline Leadership Group.

Consumers:

- Provided advice and recommendations about consumer issues and concerns related to the project as/if they occurred.

Content Experts:

- Applied their knowledge to assist the group to carry out the project to the highest standard possible.
- Provided advice on, and contributed to the groups understanding of, best practice in the areas in which they had experience and expertise.

**Guideline Leadership Group members**

| **Panel Member** | **Affiliation** |
| --- | --- |
| A/Prof Leanne Hassett (Chair, BRIDGES Chief Investigator) | - Institute for Musculoskeletal Health, Sydney Local Health District, Gadigal Country, Sydney, Australia. - Sydney School of Health Sciences, Faculty of Medicine and Health, The University of Sydney, Sydney, Australia. |
| Dr Liam Johnson (Co-chair, BRIDGES Postdoctoral Research Fellow) | - Institute for Musculoskeletal Health, Sydney Local Health District, Gadigal Country, Sydney, Australia. - School of Health Sciences, Faculty of Medicine, Dentistry and Health Sciences, University of Melbourne, Melbourne, Australia. - Physiotherapy Department, Epworth HealthCare, Melbourne, Australia. - School of Behavioural and Health Sciences, Faculty of Health Sciences, Australian Catholic University, Melbourne, Australia. |
| Professor Gavin Williams | - School of Health Sciences, Faculty of Medicine, Dentistry and Health Sciences, University of Melbourne, Melbourne, Australia. - Physiotherapy Department, Epworth HealthCare, Melbourne, Australia. |
| Professor Cathie Sherrington | - Institute for Musculoskeletal Health, Sydney Local Health District, Gadigal Country, Sydney, Australia. - Sydney School of Public Health, Faculty of Medicine and Health, The University of Sydney, Sydney, Australia. |
| A/Prof Sean Tweedy | - School of Human Movement and Nutrition Sciences, Faculty of Health and Behavioural Sciences, University of Queensland, Brisbane, Australia. - Health and Wellbeing Centre for Research Innovation, The University of Queensland, St Lucia, Australia - Queensland Centre for Olympic and Paralympic Studies, The University of Queensland, St Lucia, Australia. |
| Dr Kelly Clanchy | - School of Health Sciences and Social Work, Griffith Health, Griffith University, Gold Coast, Australia. - The Hopkins Centre, Griffith University, Nathan, Australia. |
| Professor Luke Wolfenden | - Hunter New England Local Health District, University of Newcastle, Newcastle, Australia. |
| Professor Anne Tiedemann | - Institute for Musculoskeletal Health, Sydney Local Health District, Gadigal Country, Sydney, Australia. - Sydney School of Public Health, Faculty of Medicine and Health, The University of Sydney, Sydney, Australia. |
| Professor Adrian Bauman | - Sydney School of Public Health, Faculty of Medicine and Health, The University of Sydney, Sydney, Australia. |
| Dr Catherine Carty | - Munster Technological University, UNESCO Chair Manager, Kerry, Ireland. |
| Professor Anthony Okely | - School of Health and Society, University of Wollongong, Wollongong, Australia. |
| Professor Zachary Munn | - Health Evidence Synthesis, Recommendations and Impact (HESRI), School of Public Health, Faculty of Health and Medical Sciences, University of Adelaide, Adelaide, South Australia, Australia. |
| Dr Timothy Barker | - Health Evidence Synthesis, Recommendations and Impact (HESRI), School of Public Health, Faculty of Health and Medical Sciences, University of Adelaide, Adelaide, South Australia, Australia. |
| Dr Adam Scheinberg | - Murdoch Children’s Research Institute, Melbourne, Australia |
| Ms Gabrielle Vassallo | - Consumer representative, Sydney, Australia |

BRIDGES, BRain Injury: Developing GuidElineS for physical activities; UNESCO, United Nations Educational, Scientific and Cultural Organization; HESRI, Health Evidence Synthesis, Recommendations and Impact.

**Guideline Development Group**

A Guideline Development Group was convened to develop the Australian Physical Activity Clinical Practice Guideline for people with moderate to severe TBI. The group was assembled by the chair and co-chair, with input from the Guideline Leadership Group. The purpose of the Guideline Development Group was to discuss and decide on the EtD criteria judgements contained within the guideline and provide recommendations about the clinical questions.

*In scope:*

- Decisions about the EtD criteria judgements within the clinical practice guideline.
- Decisions about the recommendations, including their wording, within the clinical practice guideline.
- Approval of the clinical practice guideline.

*Out of scope:*

- Management of the development of the clinical practice guideline. This was the responsibility of the Guideline Leadership and Guideline Steering Groups.

*Membership:*

- Membership of the Guideline Development Group included the project chief investigator and postdoctoral research fellow (chair and co-chair), a meeting chairperson, content experts across the continuum of care (including clinicians and academics), consumers with knowledge of provision of evidence-based care and representatives of stakeholder groups relevant for the guidelines (family members of people with TBI, funders and community deliverers of physical activity interventions, TBI consumer organisations, professional organisations).

A/Prof Joanne Glinsky, Macquarie University, Physiotherapist, and co-chair of the Physiotherapy Clinical Guidelines for people with spinal cord injury, facilitated the Guideline Development Group meetings. As the meeting/s facilitator, A/Prof Glinsky:

- Facilitated participation of group members.
- Managed conflicts of interest.
- Oversaw voting on EtD criteria judgements, recommendations, and their wording, contained within the clinical practice guideline.

*All members:*

- Contributed to meetings and declared relevant conflicts of interest.
- Contributed to decisions about the clinical questions contained within the clinical practice guideline.
- Voted on the EtD criteria judgements where necessary.
- Voted on recommendations, and their wording, to be contained within the clinical practice guideline.
- Contributed and provided strategies for resolution of issues with the project as/if they occurred.

*Chair and co-chair:*

- Organised pre-meeting distribution of EtD frameworks, summary of findings tables and evidence profiles for clinical questions.
- Organised workflow for tasks as required.
- Presented the draft clinical guidelines to the Guideline Development Group in the Group meetings.

*Content Experts* (Physiotherapists, exercise physiologist, academics, methodologists):

- Applied their knowledge to assist the group to carry out the project to the highest standard possible.
- Provided advice on best practice in the areas in which they had experience and expertise.
- Assisted in understanding best practice in the areas which had experience and expertise.

*Consumers:*

- Provided advice and recommendations about consumer issues and concerns related to the project as/if they occurred.

**Guideline Development Group members**

| **Panel Member (role/expertise)** | **Affiliation** |
| --- | --- |
| A/Prof Leanne Hassett (Project Chief Investigator, Physiotherapist and Academic member, clinical experience working age adults TBI, research TBI fitness & PA) | - Institute for Musculoskeletal Health, Sydney Local Health District, Gadigal Country, Sydney, Australia. - Sydney School of Health Sciences, Faculty of Medicine and Health, The University of Sydney, Sydney, Australia. |
| Dr Liam Johnson (Project Postdoctoral Research Fellow, Exercise Physiologist and Academic member, exercise prescription neurological populations, research TBI & stroke PA) | - Institute for Musculoskeletal Health, Sydney Local Health District, Gadigal Country, Sydney, Australia. - School of Health Sciences, Faculty of Medicine, Dentistry and Health Sciences, University of Melbourne, Melbourne, Australia. - Physiotherapy Department, Epworth HealthCare, Melbourne, Australia. - School of Behavioural and Health Sciences, Faculty of Health Sciences, Australian Catholic University, Melbourne, Australia. |
| Professor Gavin Williams (Physiotherapist and Academic member, clinical experience working age adults TBI, research TBI mobility & PA) | - School of Health Sciences, Faculty of Medicine, Dentistry and Health Sciences, University of Melbourne, Melbourne, Australia. - Physiotherapy Department, Epworth HealthCare, Melbourne, Australia. |
| Professor Cathie Sherrington (Physiotherapist and Academic member, research PA & mobility older adults & disability, guideline development) | - Institute for Musculoskeletal Health, Sydney Local Health District, Gadigal Country, Sydney, Australia. - Sydney School of Public Health, Faculty of Medicine and Health, The University of Sydney, Sydney, Australia. |
| A/Prof Sean Tweedy (Exercise Physiologist and Academic member, research PA & disability [high support needs], guideline development) | - School of Human Movement and Nutrition Sciences, Faculty of Health and Behavioural Sciences, University of Queensland, Brisbane, Australia. - Health and Wellbeing Centre for Research Innovation, The University of Queensland, St Lucia, Australia - Queensland Centre for Olympic and Paralympic Studies, The University of Queensland, St Lucia, Australia. |
| Dr Kelly Clanchy (Exercise Physiologist and Academic member, research acquired brain injury transition program) | - School of Health Sciences and Social Work, Griffith Health, Griffith University, Gold Coast, Australia. - The Hopkins Centre, Griffith University, Nathan, Australia. |
| Professor Luke Wolfenden (Implementation Scientist and Academic member, PA guideline development) | - Hunter New England Local Health District, University of Newcastle, Newcastle, Australia. |
| Professor Anne Tiedemann (Exercise Physiologist and Academic member, research PA and healthy ageing, WHO PA guideline development older adults) | - Institute for Musculoskeletal Health, Sydney Local Health District, Gadigal Country, Sydney, Australia. - Sydney School of Public Health, Faculty of Medicine and Health, The University of Sydney, Sydney, Australia. |
| Professor Adrian Bauman (Public Health and Academic member, PA policy and guideline development) | - Sydney School of Public Health, Faculty of Medicine and Health, The University of Sydney, Sydney, Australia. |
| Dr Catherine Carty (WHO representative and Academic member, WHO disability guidelines lead, human rights perspective) | - Munster Technological University, UNESCO Chair Manager, Kerry, Ireland. |
| Professor Anthony Okely (Methodologist and Academic member, PA guideline development) | - School of Health and Society, University of Wollongong, Wollongong, Australia. |
| Professor Zachary Munn (Methodologist member) | - Health Evidence Synthesis, Recommendations and Impact (HESRI), School of Public Health, Faculty of Health and Medical Sciences, University of Adelaide, Adelaide, South Australia, Australia. |
| Dr Tim Barker (Methodologist observer) | - Health Evidence Synthesis, Recommendations and Impact (HESRI), School of Public Health, Faculty of Health and Medical Sciences, University of Adelaide, Adelaide, South Australia, Australia. |
| Dr Adam Scheinberg (Paediatric Rehabilitation Physician member) | - Murdoch Children’s Research Institute, Melbourne, Australia |
| A/Prof Grahame Simpson (Psychology and Academic member, psychosocial rehabilitation working age adults with TBI) | - Sydney School of Health Sciences, Faculty of Medicine and Health, The University of Sydney, Sydney, Australia. Liverpool Brain Injury Rehabilitation Unit, South Western Sydney Local Health District, Sydney, Australia. - John Walsh Centre for Rehabilitation Research, Kolling Institute, Northern Sydney Local Health District and The University of Sydney, Sydney, Australia. |
| Mr Nick Rushworth (Consumer and Advocacy member) | - Brain Injury Australia, Sydney, Australia |
| Ms Gabrielle Vassallo (Consumer member, disability PA experience) | - Consumer representative, Sydney, Australia |
| Dr Abby Haynes (Academic member, qualitative research with consumers & clinicians) | - Institute for Musculoskeletal Health, Sydney Local Health District, Gadigal Country, Sydney, Australia. - Sydney School of Public Health, Faculty of Medicine and Health, The University of Sydney, Sydney, Australia. |
| Mr Rhys Ashpole (Disability funding member) | - iCare NSW, Sydney, Australia |
| Ms Sakina Chagpar (Physiotherapist member, BRIDGES guidelines steering group) | - Institute for Musculoskeletal Health, Sydney Local Health District, Gadigal Country, Sydney, Australia. |
| Ms Kerry West (Paediatric Physiotherapist member, BRIDGES guidelines steering group) | - Institute for Musculoskeletal Health, Sydney Local Health District, Gadigal Country, Sydney, Australia. - Sydney School of Public Health, Faculty of Medicine and Health, The University of Sydney, Sydney, Australia. - Physiotherapy Department, The Children’s Hospital at Westmead, Sydney, Australia. |
| Ms Belinda Wang (Exercise Physiologist member, BRIDGES guidelines steering group) | - Institute for Musculoskeletal Health, Sydney Local Health District, Gadigal Country, Sydney, Australia. - Sydney School of Public Health, Faculty of Medicine and Health, The University of Sydney, Sydney, Australia. |
| Mr Domenic Denichilo (Physiotherapist member, working in regional and remote Australia with Indigenous patients with TBI) | - Central Australia region, NT Health, Alice Springs, Australia |
| Ms Sarah Veli-Gold (Physiotherapist member, clinical experience in adult TBI, qualitative research in Indigenous population living with TBI) | - Sydney School of Health Sciences, Faculty of Medicine and Health, The University of Sydney, Sydney, Australia. |
| Mrs Sonia Hoppe (Physiotherapist member, experience community-based neurological rehabilitation including transition to community PA) | - School of Health and Rehabilitation Sciences, The University of Queensland, Brisbane, Australia |
| Ms Bhavini Whiteside (Physiotherapist member, clinical experience inpatient adult rehabilitation) | - Liverpool Hospital Brain Injury Rehabilitation Unit, South Western Sydney Local Health District, Sydney Australia |
| Mr Benjamin Sammut (Exercise Physiology student and lived-experience member) | - Consumer representative, Sydney, Australia |
| Mr Nicholas Waters (Lived-experience member) | - Consumer representative, Melbourne, Australia |
| Ms Francesca Brady (Lived-experience member) | - Consumer representative, Sydney, Australia |
| Mr Anthony Mamo (Lived-experience member) | - Consumer representative, Sydney, Australia |
| Mr Kieran Witts (Family of person with TBI) | - Consumer representative, Sydney, Australia |
| Mrs Julie Witts (Family of person with TBI) | - Consumer representative, Sydney, Australia |
| Ms Alexandra Edmonson (APA Chairperson NSW Disability Group, Physiotherapist member) | - Institute for Musculoskeletal Health, Sydney Local Health District, Gadigal Country, Sydney, Australia |
| Mr Peter Mayhew (Provider community-based disability physical activity, lived-experience member) | - Consumer representative, Perth, Australia |
| Ms Kate Heine (Head consumer organisation, advocacy, paediatric Physiotherapist member) | - Heads Together for Brain Injury, Melbourne, Australia |
| Ms Ella Bracone (Physiotherapist member, BRIDGES guidelines steering group) | - Sydney School of Public Health, Faculty of Medicine and Health, The University of Sydney, Sydney, Australia |
| Ms Sania Salim (Paediatric Physiotherapist member) | - The Royal Children’s Hospital, Melbourne, Australia |
| Ms Olivia Beattie (Paediatric Occupational Therapist member) | - The Royal Children’s Hospital, Melbourne, Australia |

TBI, Traumatic brain injury; PA, Physical activity; WHO, World Health Organisation; UNESCO, United Nations Educational, Scientific and Cultural Organization; HESRI, Health Evidence Synthesis, Recommendations and Impact; NSW, New South Wales; BRIDGES, BRain Injury: Developing GuidElineS for physical activities; NT, Northern Territory; APA, Australian physiotherapy association**.**
