# Supplementary material for: A Physical Activity Clinical Practice Guideline for People With Moderate to Severe Traumatic Brain Injury
Source: Clin Public Health Guidel. 2025 Aug 14;2(4):e70035. doi: 10.1002/gin2.70035 (PMC13563333; doi:10.1002/gin2.70035)
Supplement: Supplementary file 3 [file GIN2-2-e70035-s003.docx]

| **Outcome** | **Clinical question** | | | | | | | | | |
| --- | --- | --- | --- | --- | --- | --- | --- | --- | --- | --- |
|  | **1** | **2** | **3** | **4** | **5** | **6** | **7** | **8** | **9** | **10** |
| Cardiorespiratory fitness | C | C |  |  |  |  | I | I |  |  |
| Co-morbidities and mortality | C | C | I | I | I | I | I | I | C | C |
| Walking capacity | I | I | I | I | C | C |  |  |  |  |
| Combined mobility | I | I | C | C | C | C |  |  |  |  |
| Physical activity | I | I | I | I | I | I | I | I | C | C |
| Body composition | I | I | I | I |  |  |  |  |  |  |
| Mood | I | I |  |  |  |  | C | C | C | C |
| Muscle strength |  |  | C | C |  |  |  |  |  |  |
| Balance |  |  | I | I | C | C |  |  |  |  |
| Participation |  |  |  |  | I | I | C | C | C | C |
| Quality of life |  |  |  |  | I | I | I | I | C | C |
| Social connection |  |  |  |  |  |  | C | C | C | C |
| Behaviour change |  |  |  |  |  |  |  |  | C | C |

C, Critical; I, Important

**Clinical questions:**

1: Should **structured aerobic exercise** training compared to control be used for **adults and older adults** with moderate to severe traumatic brain injury?

2: Should **structured aerobic exercise** training compared to control be used for **children and adolescents** after moderate to severe traumatic brain injury?

3: Should **structured muscle strengthening** training compared to control be used for **adults and older adults** after moderate to severe traumatic brain injury?

4: Should **structured muscle strengthening** training compared to control be used for **children and adolescents** after moderate to severe traumatic brain injury?

5: Should **structured mobility training** (i.e., gait, balance and function training) compared to control be used for **adults and older adults** after moderate to severe traumatic brain injury?

6: Should **structured mobility training** (i.e., gait, balance and function training) compared to control be used for **children and adolescents** after moderate to severe traumatic brain injury?
7: Should **sport and physical recreation** compared to control be used for **adults and older adults** after moderate to severe traumatic brain injury?

8: Should **sport and physical recreation** compared to control be used for **children and adolescents** after moderate to severe traumatic brain injury?

9: Should **overall physical activity promotion** compared to control be used for **adults and older adults** after moderate to severe traumatic brain injury?

10: Should **overall physical activity promotion** compared to control be used for **children and adolescents** after moderate to severe traumatic brain injury?
