# Supplementary material for: A Physical Activity Clinical Practice Guideline for People With Moderate to Severe Traumatic Brain Injury
Source: Clin Public Health Guidel. 2025 Aug 14;2(4):e70035. doi: 10.1002/gin2.70035 (PMC13563333; doi:10.1002/gin2.70035)
Supplement: Supplementary file 4 [file GIN2-2-e70035-s004.docx]

| **Group Member** | **Affiliation** | **Role/Expertise** | **Declared CoI** |
| --- | --- | --- | --- |
| Leanne Hassett | - Institute for Musculoskeletal Health, Sydney Local Health District, Gadigal Country, Sydney, Australia. - Sydney School of Health Sciences, Faculty of Medicine and Health, The University of Sydney, Sydney, Australia. | - BRIDGES Chief Investigator - Physiotherapist, Academic - Clinical experience working age adults TBI, research TBI fitness & PA | - Lead investigator on the MRFF grant that has funded the development of these guidelines - Publications on work associated with this grant as Chief investigator - Published extensively on physiotherapy management after TBI - Developed educational material and given lectures and presentations on exercise rehabilitation after TBI |
| Liam Johnson | - Institute for Musculoskeletal Health, Sydney Local Health District, Gadigal Country, Sydney, Australia. - School of Health Sciences, Faculty of Medicine, Dentistry and Health Sciences, University of Melbourne, Melbourne, Australia. - Physiotherapy Department, Epworth HealthCare, Melbourne, Australia. - School of Behavioural and Health Sciences, Faculty of Health Sciences, Australian Catholic University, Melbourne, Australia. | - BRIDGES Postdoctoral Research Fellow - Exercise Physiologist, Academic - Exercise prescription neurological population, research TBI & stroke PA | - Postdoctoral Fellow on the BRIDGES project - Publication on work associated with this grant as lead author - Published extensively on exercise rehabilitation and PA in stroke |
| Gavin Williams | - School of Health Sciences, Faculty of Medicine, Dentistry and Health Sciences, University of Melbourne, Melbourne, Australia. - Physiotherapy Department, Epworth HealthCare, Melbourne, Australia. | - BRIDGES Investigator - Physiotherapist, Academic - Clinical experience working age adults TBI, research TBI mobility & PA | - Has published journal articles on TBI exercise rehabilitation - Has developed educational material and given lectures and presentations on exercise rehabilitation after TBI |
| Cathie Sherrington | - Institute for Musculoskeletal Health, Sydney Local Health District, Gadigal Country, Sydney, Australia. - Sydney School of Public Health, Faculty of Medicine and Health, The University of Sydney, Sydney, Australia. | - BRIDGES Investigator - Physiotherapist, Academic - Research PA & mobility older adults & disability, guideline development | - Has published journal articles on exercise/rehabilitation - Has given lectures and presentations on exercise/rehabilitation - Has various media appearances on falls in older adults and PA - Serves on voluntary roles as Secretary, Cerebral Palsy Sport and Recreation Association of NSW and Board Member, World Abilitysport |
| Sean Tweedy | - School of Human Movement and Nutrition Sciences, Faculty of Health and Behavioural Sciences, University of Queensland, Brisbane, Australia. - Health and Wellbeing Centre for Research Innovation, The University of Queensland, St Lucia, Australia - Queensland Centre for Olympic and Paralympic Studies, The University of Queensland, St Lucia, Australia. | - BRIDGES Investigator - Exercise Physiologist, Academic - Research PA & disability (high support needs), guideline development | - Has published factsheet on ABI exercise - Has developed educational material and given lectures on exercise/rehabilitation |
| Kelly Clanchy | - School of Health Sciences and Social Work, Griffith Health, Griffith University, Gold Coast, Australia. - The Hopkins Centre, Griffith University, Nathan, Australia. | - BRIDGES Investigator - Exercise Physiologist, Academic - Research ABI transition program | - Received grants to evaluate interventions promoting PA in individuals with a disability. No funding bodies were involved with collation, analysis or reporting of results - Has published journal articles on PA participation in adults with TBI |
| Luke Wolfenden | - Hunter New England Local Health District, University of Newcastle, Newcastle, Australia. | - BRIDGES Investigator, - Public Health, Implementation Scientist, Academic - Implementation science, PA, guideline development | - Nothing to declare |
| Anne Tiedemann | - Institute for Musculoskeletal Health, Sydney Local Health District, Gadigal Country, Sydney, Australia. - Sydney School of Public Health, Faculty of Medicine and Health, The University of Sydney, Sydney, Australia. | - BRIDGES Investigator, - Exercise Physiologist, Academic - Research PA & healthy ageing, WHO guideline development older adults | - Nothing to declare |
| Adrian Bauman | - Sydney School of Public Health, Faculty of Medicine and Health, The University of Sydney, Sydney, Australia. | - BRIDGES Investigator, - Public Health researcher, Academic - Research public health & PA, policy, guideline development | - Nothing to declare |
| Catherine Carty | - Munster Technological University, UNESCO Chair Manager, Kerry, Ireland | - WHO Representative, UNESCO Chair Manager - WHO disability guidelines lead, human rights perspective | - Mentioned in lectures that guidelines are under development |
| Anthony Okely | - School of Health and Society, University of Wollongong, Wollongong, Australia. | - Methodologist - PA, guideline development using ADOLOPMENT process | - Publications, lectures and media appearances in relation to the 24-hour movement Guidelines for Australia for the Early Years and for Children and Young people - Current chair of the Leadership group for updating the above mentioned guidelines |
| Zachary Munn | - Health Evidence Synthesis, Recommendations and Impact (HESRI), School of Public Health, Faculty of Health and Medical Sciences, University of Adelaide, Adelaide, South Australia, Australia. | - Methodologist - Guideline development, GRADE process | - Chair of the Guidelines International network - GRADE Centre Director - Member of NHMRC Synthesis and Translation of Research Evidence - GRADE Institute received funding for me to act as a methodologist for this guideline |
| Adam Scheinberg | - Murdoch Children’s Research Institute, Melbourne, Australia | - BRIDGES Investigator - Rehabilitation Specialist, Academic - Paediatrics Rehabilitation Specialist ABI | - Publications on work associated with this grant as an Associate Investigator |
| Grahame Simpson | - Sydney School of Health Sciences, Faculty of Medicine and Health, The University of Sydney, Sydney, Australia. Liverpool Brain Injury Rehabilitation Unit, South Western Sydney Local Health District, Sydney, Australia. - John Walsh Centre for Rehabilitation Research, Kolling Institute, Northern Sydney Local Health District and The University of Sydney, Sydney, Australia. | - BRIDGES Investigator - Social Worker, Psychologist, Academic - Psychosocial rehabilitation working age adults TBI | - Publications on work associated with this grant as an Associate Investigator |
| Nick Rushworth | - Brain Injury Australia, Sydney, Australia | - BRIDGES Investigator - TBI Consumer, lead of Representative body - Brain Injury advocacy, TBI Mission | - Nothing to declare |
| Gabrielle Vassallo | - Consumer representative, Sydney, Australia | - BRIDGES Investigator - TBI Consumer - Brain Injury lived experience, PA participation | - Publications on work associated with this grant as an Associate Investigator - Associate Investigator on the MRFF grant that has funded the development of these guidelines |
| Abby Haynes | - Institute for Musculoskeletal Health, Sydney Local Health District, Gadigal Country, Sydney, Australia. - Sydney School of Public Health, Faculty of Medicine and Health, The University of Sydney, Sydney, Australia. | - BRIDGES Investigator - Qualitative health researcher - BRIDGES qualitative data, qualitative research with consumers & clinicians | - Many publications on physical activity - Publication on how to conduct research to inform a DCE that will inform these guidelines |
| Rhys Ashpole | - iCare NSW, Sydney, Australia | - BRIDGES Partner - Funder icare NSW - State-based funding for TBI injury | - Nothing to declare |
| Sakina Chagpar | - Institute for Musculoskeletal Health, Sydney Local Health District, Gadigal Country, Sydney, Australia. | - BRIDGES Research Officer - Physiotherapist - BRIDGES dataset, research neurological rehabilitation | - Publications on work associated with the grant developing this guideline |
| Kerry West | - Institute for Musculoskeletal Health, Sydney Local Health District, Gadigal Country, Sydney, Australia. - Sydney School of Public Health, Faculty of Medicine and Health, The University of Sydney, Sydney, Australia. - Physiotherapy Department, The Children’s Hospital at Westmead, Sydney, Australia. | - BRIDGES Research Officer - Physiotherapist - BRIDGES qualitative data, paediatric physiotherapy public hospital services | - Nothing to declare |
| Belinda Wang | - Institute for Musculoskeletal Health, Sydney Local Health District, Gadigal Country, Sydney, Australia. - Sydney School of Public Health, Faculty of Medicine and Health, The University of Sydney, Sydney, Australia. | - BRIDGES Research Assistant - Exercise Physiologist - BRIDGES dataset, clinical experience working with adults with TBI | - Nothing to declare |
| Domenic Denichilo | - Central Australia region, NT Health, Alice Springs, Australia. | - Physiotherapist - Working with Indigenous populations in central Australia | - Nothing to declare |
| Sarah Veli-Gold | - Sydney School of Health Sciences, Faculty of Medicine and Health, The University of Sydney, Sydney, Australia. | - Physiotherapist - Clinical experience in adult TBI, qualitative research in Indigenous populations living with TBI | - Employed at inner city hospital advocating for physiotherapy within the neurological rehabilitation setting |
| Sonia Hoppe | - School of Health and Rehabilitation Sciences, The University of Queensland, Brisbane, Australia. | - Physiotherapist - Clinical experience with adults living in the community (private/public) | - Developed education material for physiotherapy students on clinical placement |
| Bhavini Whiteside | - Liverpool Hospital Brain Injury Rehabilitation Unit, South Western Sydney Local Health District, Sydney Australia | - Physiotherapist - Clinical experience inpatient rehabilitation | - Nothing to declare |
| Benjamin Sammut | - Consumer representative | - Consumer- young adult - Injury as adolescent/young adult. Recent Exercise Physiologist graduate | - Nothing to declare |
| Nicholas Waters | - Consumer representative, Melbourne, Australia | - Consumer- adult - 36-45; Injury 2-5 yrs ago, 3-12 months in hospital, currently active | - Nothing to declare |
| Francesca Brady | - Consumer representative | - Consumer- older adult - 56-65; injury 2-5 yrs ago, <2wks in hospital, inactive, living regionally | - Nothing to declare |
| Anthony Mamo | - Consumer representative, Sydney, Australia | - Consumer / person with TBI | - Receives NDIS funding for disability - Has lectured on lived experience with TBI to physiotherapy students |
| Kieran Witts | - Consumer representative | - Family member person with TBI - Parents of adult/child with severe TBI (living regional) | - Nothing to declare |
| Julie Witts | - Consumer representative | - Family member person with TBI - Parents of adult/child with severe TBI (living regional) | - Nothing to declare |
| Alexandra Edmonson | - Institute for Musculoskeletal Health, Sydney Local Health District, Gadigal Country, Sydney, Australia | - NSW lead disability group, APA - Policy & Advocacy | - Nothing to declare |
| Peter Mayhew | - Consumer representative, Perth, Australia | - Community disability gym owner - Delivering community-based PA to people with TBI/lived experience (TBI only strengthening gym) | - Run’s a not-for-profit community fitness facility that supports people rehabilitation from injury or trauma include stroke and ABI (TBI) |
| Kate Heine | - Heads Together for Brain Injury, Melbourne, Australia | - Representative Consumer organisation - Support and advocacy for children & adolescents & families living with ABI | - Nothing to declare |
| Ella Bracone | - Sydney School of Public Health, Faculty of Medicine and Health, The University of Sydney, Sydney, Australia | - Physiotherapist/research team | - Nothing to declare |
| Joanne Glinsky | - Sydney School of Health Sciences, Faculty of Medicine and Health, The University of Sydney, Sydney, Australia. - John Walsh Centre for Rehabilitation Research, Kolling Institute, Northern Sydney Local Health District and The University of Sydney, Sydney, Australia | - Facilitator | - Have received research grants from iCare NSW - Lectured students in relation to TBI |
| Sania Salim | - The Royal Children’s Hospital, Melbourne, Australia | - Paediatric physiotherapist - Paediatrics Rehabilitation Specialist ABI | - Nothing to declare |
| Olivia Beattie | - The Royal Children’s Hospital, Melbourne, Australia | - Paediatric Occupational Therapist - Paediatrics Rehabilitation Specialist ABI | - Nothing to declare |

CoI, Conflict of interest; BRIDGES, BRain Injury: Developing GuidElineS for physical activities; TBI, Traumatic brain injury; PA, Physical activity; MRFF, Medical Research Future Fund; NSW, New South Wales; ABI, Acquired brain injury; WHO, World Health Organisation; UNESCO, United Nations Educational, Scientific and Cultural Organization; GRADE, Grading of Recommendations Assessment, Development and Evaluation; NHMRC, National Health and Medical Research Council; DCE, Discrete choice experiment; NT, Northern Territory; NDIS, National Disability Insurance Scheme; APA, Australian physiotherapy association.
