# Supplementary material for: A Physical Activity Clinical Practice Guideline for People With Moderate to Severe Traumatic Brain Injury
Source: Clin Public Health Guidel. 2025 Aug 14;2(4):e70035. doi: 10.1002/gin2.70035 (PMC13563333; doi:10.1002/gin2.70035)
Supplement: Supplementary file 5 [file GIN2-2-e70035-s005.docx]

**Australian Physical Activity Clinical Practice Guideline for people with moderate to severe traumatic brain injury**

**Conflict of Interest Policy**

**Scope of this policy**

This conflict of interest policy is to minimise potential conflicts biasing the recommendations in the Australian Physical Activity Clinical Practice Guideline for people with moderate to severe traumatic brain injury (TBI). It applies to the Guideline Steering Group, the Guideline Leadership Group, and the Guideline Development Group, and any contributors to, or reviewers of, the guideline.

**Definition**

A conflict of interest may occur from any direct or indirect financial or non-financial interest. Non-financial interests can include but is not limited to publishing research that may be used in the guideline, being considered an opinion leader and having personal experience of a disease.

**Process for appointing chairs and the Guideline Development Group**

The chair/s of the Guideline Development Group is appointed by the Chief Investigator of the Project/Grant. Should the chair resign the Chief Investigator will appoint a new chair. Guideline Development Group members are representative of as many areas of TBI care as possible (i.e., involving key stakeholders, across the continuum of care, representing children, adolescents, adults and older adults, and across the states and territories of Australia). Groups representative of areas of care will be approached and asked for a representative/s. The representative will be chosen by the service/area of care. Reviewers of the guideline will be suggested by the Guideline Leadership Group and appointed by the National Health and Medical Research Council Clinical Guidelines group.

**Guidelines for accepting sponsorship**

The Australian Physical Activity Clinical Practice Guideline for people with moderate to severe TBI is funded by the Medical Research Future Fund (MRFF) 2020 Traumatic Brain Injury-Mission (MRF2009099). Further funding may be received from government, hospital or association grants. No funding will be accepted from a commercial enterprise that has an interest in the outcome of the guideline.

**Process for identifying conflicts of interest**

The conflict of interest policy will be given to all members of the Guideline Steering, Leadership, and Development Groups, and reviewers. All parties will be explicitly asked by the chair if they have any conflicts of interest. If conflicts are known but not declared, the chair will contact the individual involved for clarification. The conflict of interest process is ongoing over the course of the project. If conflicts arise members should update their status.

**Process for disclosing interests**

A conflict of interest can be declared in group meetings or via email to the chair. If a conflict of interest is declared in a committee meeting it will be recorded in the minutes. If a conflict of interest is emailed to the chair it will be declared and placed in the minutes at the next committee meeting.

**Process for managing conflicts of interest**

1. Identify conflicts of interest at the beginning of and throughout the process of guideline development.
2. Record conflict of interest and provide this information within the final guideline.
3. Exclude any representation of the funding body from the clinical practice Guideline Development Group.
4. Implement an anonymous voting process for all decisions related to the recommendations.
5. Record the results or the votes for each recommendation. Declare the number included in the vote and the result for each recommendation.
6. Exclude authors from voting where their publications constitute the only evidence on a recommendation.
7. Exclude group members from the Guideline Development Group for failing to disclose a major conflict of interest.

**Additional Note**

This conflict of interest policy was adapted from the Physiotherapy Clinical Practice Guidelines for people with Spinal Cord Injury, which was based on the 2014 UK National Institute for Health and Care Excellence (NICE) Policy on Conflicts of Interest.
