# Supplementary material for: A Physical Activity Clinical Practice Guideline for People With Moderate to Severe Traumatic Brain Injury
Source: Clin Public Health Guidel. 2025 Aug 14;2(4):e70035. doi: 10.1002/gin2.70035 (PMC13563333; doi:10.1002/gin2.70035)
Supplement: Supplementary file 6 [file GIN2-2-e70035-s006.docx]

**Literature searches and evidence eligibility**

*Aim of the systematic review evidence to inform the Guideline*

A systematic review was conducted to determine the effectiveness of physical activity interventions on measures of physical function, cognition, and quality of life (primary objectives); physical activity, participation, comorbidities and mortality, mood, behaviour change, pain, and social connection (secondary objectives) in people with moderate to severe TBI.

*Types of studies*

Published randomised controlled trials (RCTs, including cross-over RCTs) and non-randomised studies of interventions (NRSIs) were included. Trials with more than two parallel comparisons were included if two of the comparisons met the inclusion criteria. If trials were reported in more than one publication or interim analyses were published prior to the completion of the trial, then the most recent publication was used. Only trials published in English were included.

*Types of participants*

Children, adolescents, adults and older adults with moderate to severe TBI were included. Trials with a mix of participants with different neurological conditions (i.e., stroke, Parkinson’s disease) were also included.

*Types of interventions*

We included studies of any physical activity intervention that would contribute to the person meeting the WHO physical activity guideline definition of physical activity i.e., *'any activity involving bodily movement produced by skeletal muscles that requires energy expenditure'*.^1^ This included: structured aerobic training, structured muscle strengthening, structured gait/balance/functional training, sport and physical recreation, and promotion of physical activity. The intervention may be delivered as a standalone intervention or as part of a rehabilitation package. The intervention may be implemented as an inpatient, outpatient or community-based program, may be supervised or self-led, and be set in a health, home, work, school or community setting. Given the inclusion of rehabilitation-based studies, where interventions incorporated robotics that passively assisted movement, only studies where the intervention required the participant to produce at least 50% voluntary/unassisted activity were included.

*Types of comparisons*

RCTs and NRSIs were included if they compared the interventions of interest with i) usual care, ii) a physical activity intervention with different parameters (i.e., dose, setting, or supervision), iii) a non-physical activity intervention, or iv) no intervention. Trials that compared interventions with an alternate intervention were also included if they were a PICO of interest. Trials that included a co-intervention or usual care were included if the co-intervention or usual care were administered to both groups (making it possible to determine the added benefit of the intervention of interest).

*Types of outcome measures*

Trials were included that contained an outcome relevant to the PICO. These included measures of physical function (i.e., cardiorespiratory fitness, mobility, balance, muscle strength, body composition, walking capacity, fatigue), cognition, quality of life, comorbidities and mortality, mood, participation, physical activity, behaviour change, pain, and social connection.

*Search methods for identification of studies*

The following electronic databases were searched to identify reports of relevant studies:

Ovid MEDLINE (1946 to December 24th 2022); Ovid CENTRAL (1991 to December 24^th^ 2022) and EBSCO SPORTDiscus (searched December 24th 2022).

To search these three databases, search terms for RCTs, systematic reviews and meta-analyses, and NRSIs, were combined with search terms for physical activity and TBI. See Appendix 7 for full search strategies.

*Searching other resources*

The reference lists of all identified systematic reviews were also searched.

*Selection of studies*

11,400 articles were initially imported into Endnote before duplicates were removed and the remaining records were imported into a web-based data management platform (Covidence 2020 v1517, Melbourne, Australia) for screening. Using the aforementioned eligibility criteria, a team of five reviewers completed the title and abstract screening of 11,382 articles. Two reviewers independently screened all records, with conflict resolution completed by a third reviewer (LJ). The same team of reviewers completed the full text screening of 213 articles. Each full text record was screened by two reviewers independently, with studies excluded based on the exclusion criteria. Conflict resolution was completed by a third reviewer (LJ). In total, 128 articles were included in the review (See Figure 1 for flow of records). One reviewer then matched the selected studies to each clinical question.

**Evidence synthesis methods**

*Data extraction and management*

Data extraction was completed by a single reviewer using a self-developed, customised data extraction template in a Microsoft Excel spreadsheet. The data extraction form was developed and piloted on two studies by two reviewers (PA and BW). Data extraction included information on:

• study design, setting, and location

• sample size and sample characteristics (i.e., age, gender, injury severity)

• intervention components (i.e., frequency, intensity, time and type)

• comparison intervention

• outcome measures

• key findings

In instances of mixed study populations (i.e., mild, moderate and severe TBI, TBI and other acquired brain injuries), where possible, only moderate to severe TBI data were extracted. If this was not possible, group data was used in the synthesis and analysis. Where multiple measures were used in a single study to assess the same, or similar, construct, the authors chose the measure they believed most appropriately measured the construct given their experience in the field and knowledge of the literature.

*Details of data extraction for synthesis*

Two authors independently extracted data from each study to determine between-group mean differences (MD) and 95% confidence intervals (95% CI). This included outcome scores and number of participants overall and in each group. If results were only presented graphically, the mean scores and standard deviation (SD) were estimated from the figures provided using WebPlotDigitizer to extract numerical data.^2^ The following rules were used (from first to last) when deciding upon which data to extract:

• mean between-group differences in post-intervention scores.

• mean and SD of change scores provided in the studies (post-intervention scores and change scores were not pooled in meta-analyses in which results were expressed as standardised mean differences (SMD)).

• mean (SD) post-intervention scores.

Where median and interquartile range (IQR) were reported, the mean and SD were calculated as per the quantile estimation method described by McGrath et al. (2020).^3^ First period data only was extracted if possible from cross-over RCTs. RevMan 5.4.1 software was used to convert 95% CI, standard errors, p values and any other appropriate combination of data or statistical results into SDs when necessary. The direction of effect of each outcome was standardised.

Meta-analyses were conducted across studies that made similar comparisons if there were at least two studies without excessive clinical heterogeneity. Clinical heterogeneity was assessed by examining the type of participants, type and intensity of the intervention, and other issues related to the design and conduct of the studies. If studies in a meta-analysis used the same measure and same units, effects were expressed as MD and 95% CI. If different measures or different units were used within a meta-analysis, effects were expressed as SMD and 95% CI. In calculating SMD, post-intervention scores were not pooled with change scores. Data were analysed using RevMan v5.4.1. A lack of data meant no sub-group or sensitivity analysis were performed.

*Dealing with missing data*

All feasible available results were included. Only published data was extracted to use in analysis. All available data were converted where possible (for example, when data were reported as standard errors) using the calculator incorporated into RevMan.

*Assessment of heterogeneity*

Heterogeneity was determined by visual inspection of the forest plots and with consideration of the I^2^ test. We did not test for publication bias due to the small number of studies included in the meta-analysis.

*Assessment of risk of bias in included studies*

We assessed the risk of bias in each trial using Cochrane risk of bias (RoB) tools. For RCTs and cross-over RCTs, the RoB-2 was used,^4^ while for NRSI, the ROBINS-I instrument was used.^5^ For all studies, a single reviewer independently assessed the domains of potential bias arising for each domain of the relevant tool. The level of potential bias was judged as low, high, or unclear (due to a lack of information or uncertainty) for each domain. For measures of treatment effect for outcomes measured on the same scale, we calculated the MD and 95% CI using a random-effects model. Where outcomes were measured using different measures, we calculated the SMD (Hedges’ g) and 95% CI using a random-effects model to pool estimates. Data were pooled in meta-analyses where appropriate and reasonable. Where change scores were reported, these were pooled with end of intervention and/or end of follow-up scores for analysis but are presented for these studies as separate subgroups.^6^ Effect sizes were categorised as small (0.1 to 0.4), medium (0.5 to 0.7), or large (0.8 or greater). Where it was not possible or appropriate to pool data, study results were narratively synthesised.

^1^ Carty C, van der Ploeg HP, Biddle SJH, Bull F, Willumsen J, Lee L, et al. The first global physical activity and sedentary behavior guidelines for people living with disability. J Phys Act Health [Internet]. 2021 [cited 2024 Sep 22]; 18(1). DOI: 10.1123/jpah.2020-0629.

^2^ Rohatgi A. WebPlotDigitizer (Version 4.6) [Internet]. Pacifica, California USA. 2022. [cited 2024 Sept 22]. Available from https://automeris.io/WebPlotDigitizer.

^3^ McGrath S, Zhao X, Steele R, Thombs BD, Benedetti A; DEPRESsion Screening Data (DEPRESSD) Collaboration. Estimating the sample mean and standard deviation from commonly reported quantiles in meta-analysis. Stat Methods Med Res [Internet]. 2020 [cited 2024 Sep 23]; 29(9). DOI: 10.1177/0962280219889080.

^4^ Sterne JAC, Savović J, Page MJ, Elbers RG, Blencowe NS, Boutron I, et al. RoB 2: a revised tool for assessing risk of bias in randomised trials. BMJ [Internet]. 2019 [cited 2024 Sep 23]; 366. DOI: 10.1136/bmj.l4898.

^5^ Sterne JA, Hernán MA, Reeves BC, Savović J, Berkman ND, Viswanathan M, et al. ROBINS-I: a tool for assessing risk of bias in non-randomised studies of interventions BMJ [Internet]. 2016 [cited 2024 Sep 23]; 355. DOI:10.1136/bmj.i4919.

^6^ Deeks et al., 2022 Deeks JJ, Higgins JPT, Altman DG (editors). Chapter 10: Analysing data and undertaking meta-analyses. In: Higgins JPT, Thomas J, Chandler J, Cumpston M, Li T, Page MJ, Welch VA (editors). Cochrane Handbook for Systematic Reviews of Interventions version 6.3 (updated 2022 Feb). Cochrane [Internet]. 2022. [cited 2024 Sep 23]. Available from www.training.cochrane.org/handbook.
