# Supplementary material for: A Physical Activity Clinical Practice Guideline for People With Moderate to Severe Traumatic Brain Injury
Source: Clin Public Health Guidel. 2025 Aug 14;2(4):e70035. doi: 10.1002/gin2.70035 (PMC13563333; doi:10.1002/gin2.70035)
Supplement: Supplementary file 7 [file GIN2-2-e70035-s007.docx]

**CENTRAL**

1.	exp Craniocerebral Trauma/

2.	Craniocerebral Trauma*.mp.

3.	exp Brain Injuries, Traumatic/

4.	exp Diffuse Axonal Injury/

5.	diffus* axonal injur*.mp.

6.	exp Brain Injuries, Diffuse/

7.	((head or crani* or capitis or brain* or forebrain* or skull* or hemisphere or intracran* or orbit* or cerebr*) adj1 (injur* or trauma* or lesion* or damage* or wound* or destruction* or oedema* or edema* or fracture* or contusion* or commotion* or pressur*)).ti,ab,kw.

8.	Diffus* brain injur*.mp.

9.	TBI.ti,ab,kw.

10.	exp Brain Injuries/

11.	1 or 2 or 3 or 4 or 5 or 6 or 7 or 8 or 9 or 10

12.	exp Exercise/

13.	Exercis*.mp.

14.	Physical activit*.mp.

15.	exp Exercise Therapy/

16.	exp Physical Fitness/

17.	Physical fitness*.mp.

18.	exp Sports/

19.	Sport*.mp.

20.	exp Physical Exertion/

21.	physical exertion.mp.

22.	exp Physical Therapy Modalities/

23.	Physical therap*.mp.

24.	exp Resistance Training/

25.	Resistance train*.mp.

26.	Physiotherap*.mp.

27.	((exercis* or circuit or aerobic or cardio* or musc* or weight* or strength* or resistance or balance or endurance or treadmill or motor* or power* or task* or mobility or gait or fitness or physical*) adj1 (therap* or train* or retrain* or program* or intervention* or protocol* or activit* or regim* or group* or class*)).tw,ti,ab.

28.	(Baseball or Basketball or Bicycl* or Boxing or Football or Golf or Gymnastics or Hockey or dance* or racquet Sport* or cricket* or team sport* or run* or skat* or snow sport* or soccer* or swim* or mountain bik* or AFL or alpine ski* or archery or athletic* or badminton or basketball or biathlon or biking or Boxing or canoe* or cricket or cross country ski* or curling or cycl* or diving or duathlon or equestrian or fencing or football or golf or gymnastics or Handball or hippotherapy or Hockey or horseback riding or horse riding or judo or kayak or kickboxing or lawn bowls or bowling or marathon or netball or badminton or snowboard or triathlon or Polo or powerlifting or rowing or sailing or shooting or skiing or snowboard or soccer or surfing or table tennis or taekwondo or Tae Kwon Do or tenpin bowling or Tennis or Trampolin* or triathlon or volleyball or volley or australian football or baseball or fencing or racing or rugby or sport* or tennis or union or league or Yoga or Tai chi or Tai ji or Chi kung or Qiqong or stretching).tw,ti,ab.

29.	exp Water Sports/

30.	exp Racquet Sports/

31.	exp Snow Sports/

32.	exp Team Sports/

33.	exp Return to Sport/

34.	return to sport.mp.

35.	12 or 13 or 14 or 15 or 16 or 17 or 18 or 19 or 20 or 21 or 22 or 23 or 24 or 25 or 26 or 27 or 28 or 29 or 30 or 31 or 32 or 33 or 34

36.	11 and 35

37.	exp Placebos/

38.	Random Assignment*.mp.

39.	control groups/

40.	cross-over studies/

41.	(crossover or cross-over or cross over).tw.

42.	(random* or placebo* or "clinical trial*").mp.

43.	((singl* or doubl* or trebl* or tripl*) adj1 (blind* or mask*)).ab,ti.

44.	Systematic review*.mp.

45.	exp Randomized Controlled Trials as Topic/

46.	(randomized controlled trial or controlled clinical trial or placebo*).tw,pt.

47.	exp Cohort Studies/

48.	evaluation studies as topic/ or feasibility studies/ or pilot projects.mp. [mp=title, book title, abstract, original title, name of substance word, subject heading word, floating sub-heading word, keyword heading word, organism supplementary concept word, protocol supplementary concept word, rare disease supplementary concept word, unique identifier, synonyms]

49.	((control and group*) or study or cohort or comparative stud*or evaluation studies).mp.

50.	comparative study/ or meta-analysis/

51.	(comparative stud* or meta-analysis*).mp.

52.	37 or 38 or 39 or 40 or 41 or 42 or 43 or 44 or 45 or 46 or 47 or 48 or 49 or 50 or 51

53.	36 and 52

54.	53 and "Humans".sa_suba.

**MEDLINE**

1.	exp Craniocerebral Trauma/

2.	Craniocerebral Trauma*.mp.

3.	exp Brain Injuries, Traumatic/

4.	exp Diffuse Axonal Injury/

5.	diffus* axonal injur*.mp.

6.	exp Brain Injuries, Diffuse/

7.	((head or crani* or capitis or brain* or forebrain* or skull* or hemisphere or intracran* or orbit* or cerebr*) adj1 (injur* or trauma* or lesion* or damage* or wound* or destruction* or oedema* or edema* or fracture* or contusion* or commotion* or pressur*)).ti,ab,kw.

8.	Diffus* brain injur*.mp.

9.	TBI.ti,ab,kw.

10.	exp Brain Injuries/

11.	1 or 2 or 3 or 4 or 5 or 6 or 7 or 8 or 9 or 10

12.	exp Exercise/

13.	Exercis*.mp.

14.	Physical activit*.mp.

15.	exp Exercise Therapy/

16.	exp Physical Fitness/

17.	Physical fitness*.mp.

18.	exp Sports/

19.	Sport*.mp.

20.	exp Physical Exertion/

21.	physical exertion.mp.

22.	exp Physical Therapy Modalities/

23.	Physical therap*.mp.

24.	exp Resistance Training/

25.	Resistance train*.mp.

26.	Physiotherap*.mp.

27.	((exercis* or circuit or aerobic or cardio* or musc* or weight* or strength* or resistance or balance or endurance or treadmill or motor* or power* or task* or mobility or gait or fitness or physical*) adj1 (therap* or train* or retrain* or program* or intervention* or protocol* or activit* or regim* or group* or class*)).tw,ti,ab.

28.	(Baseball or Basketball or Bicycl* or Boxing or Football or Golf or Gymnastics or Hockey or dance* or racquet Sport* or cricket* or team sport* or run* or skat* or snow sport* or soccer* or swim* or mountain bik* or AFL or alpine ski* or archery or athletic* or badminton or basketball or biathlon or biking or Boxing or canoe* or cricket or cross country ski* or curling or cycl* or diving or duathlon or equestrian or fencing or football or golf or gymnastics or Handball or hippotherapy or Hockey or horseback riding or horse riding or judo or kayak or kickboxing or lawn bowls or bowling or marathon or netball or badminton or snowboard or triathlon or Polo or powerlifting or rowing or sailing or shooting or skiing or snowboard or soccer or surfing or table tennis or taekwondo or Tae Kwon Do or tenpin bowling or Tennis or Trampolin* or triathlon or volleyball or volley or australian football or baseball or fencing or racing or rugby or sport* or tennis or union or league or Yoga or Tai chi or Tai ji or Chi kung or Qiqong or stretching).tw,ti,ab.

29.	exp Water Sports/

30.	exp Racquet Sports/

31.	exp Snow Sports/

32.	exp Team Sports/

33.	exp Return to Sport/

34.	return to sport.mp.

35.	12 or 13 or 14 or 15 or 16 or 17 or 18 or 19 or 20 or 21 or 22 or 23 or 24 or 25 or 26 or 27 or 28 or 29 or 30 or 31 or 32 or 33 or 34

36.	11 and 35

37.	exp Placebos/

38.	Random Assignment*.mp.

39.	control groups/

40.	cross-over studies/

41.	(crossover or cross-over or cross over).tw.

42.	(random* or placebo* or "clinical trial*").mp.

43.	((singl* or doubl* or trebl* or tripl*) adj1 (blind* or mask*)).ab,ti.

44.	Systematic review*.mp.

45.	exp Randomized Controlled Trials as Topic/

46.	(randomized controlled trial or controlled clinical trial or placebo*).tw,pt.

47.	exp Cohort Studies/

48.	evaluation studies as topic/ or feasibility studies/ or pilot projects.mp. [mp=title, book title, abstract, original title, name of substance word, subject heading word, floating sub-heading word, keyword heading word, organism supplementary concept word, protocol supplementary concept word, rare disease supplementary concept word, unique identifier, synonyms]

49.	((control and group*) or study or cohort or comparative stud*or evaluation studies).mp.

50.	comparative study/ or meta-analysis/

51.	(comparative stud* or meta-analysis*).mp.

52.	37 or 38 or 39 or 40 or 41 or 42 or 43 or 44 or 45 or 46 or 47 or 48 or 49 or 50 or 51

53.	36 and 52

54.	53 and "Humans".sa_suba.

**SPORTDiscus**

S1	"BRAIN damage" OR "BRAIN injuries" OR "BRAIN damage" OR "CHRONIC traumatic encephalopathy"

S2	"diffus* axonal injur*"

S3	"diffus* brain injur*"

S4	((“head” OR “crani*” OR “capitis” or “brain*” OR “forebrain*” OR “skull*” OR “hemisphere” OR “intracran*” OR “orbit*” OR “cerebr*”) N1 (“injur*” OR “trauma*” OR “lesion*” or “damage*” OR “wound*” OR “destruction*” OR “oedema*” OR “edema*” OR “fracture*” OR “contusion*” OR “commotion*” OR “pressur*”) )

S5	TI "TBI" OR AB "TBI"

S6	S1 OR S2 OR S3 OR S4 OR S5

S7	exercis*

S8	"Physical activit*"

S9	"exercise therap*"

S10	"physical fitness"

S11	Sport*

S12	“recreational therap*”

S13	"Resistance train*"

S14	( (“exercis*” OR “circuit” OR “aerobic” OR “cardio*” OR “musc*” OR “weight*” OR “strength*” OR “resistance” OR “balance” OR “endurance” OR “treadmill” OR “motor*” OR “power*” OR “task*” OR “mobility” OR “gait” OR “fitness” OR “physical*”) N1 (“therap*” OR “train*” OR “retrain*” OR “program*” OR “intervention*” OR “protocol*” OR “activit*” OR “regim*” OR “group*” OR “class*”) )

S15	"physiotherap*"

S16	( “Baseball” OR “Basketball” OR “Bicycl*” OR “Boxing” OR “Football” OR “Golf” OR “Gymnastics” OR “Hockey” OR “dance*” OR “racquet Sport*” OR “cricket*” OR “team sport*” OR “run*” OR “skat*” OR “snow sport*” OR “soccer*” OR “swim*” OR “mountain bik*” OR “AFL” OR “alpine ski*” OR “archery” OR “athletic*” OR “badminton” OR “basketball” OR “biathlon” OR “biking” OR “Boxing” OR “canoe*” OR “cricket” OR “cross country ski*” OR “curling” OR “cycl*” OR “diving” OR “duathlon” OR “equestrian” OR “fencing” OR “football” OR “golf” OR “gymnastics” OR “Handball” OR “hippotherapy” OR “Hockey” OR “horseback riding” OR “horse riding” OR “judo” OR “kayak” OR “kickboxing” OR “lawn bowls” OR “bowling” OR “marathon” OR “netball” OR “badminton” OR “snowboard” OR “triathlon” OR “Polo” OR “powerlifting” OR “rowing” OR “sailing” OR “shooting” OR “skiing” OR “snowboard*” OR “soccer” OR “surfing” OR “table tennis” OR “taekwondo” OR “Tae Kwon Do” OR “tenpin bowling” OR “Tennis” OR “Trampolin*” OR “triathlon” OR “volleyball” OR “volley” OR “australian football” OR “baseball” OR “fencing” OR “racing” OR “rugby” OR “sport*” OR “tennis” OR “union “OR “league” OR “Yoga” OR “Tai chi” OR “Tai ji” OR “Chi kung” OR “Qiqong” OR “stretching” or "team sport* or "ball game*" or "Aquatic sport*" or "water sport*" or "racquet sport*" or "snow sport*" or "team sport*" or "return to sport*" )

S17	hydrotherap*

S18	S7 OR S8 OR S9 OR S10 OR S11 OR S12 OR S13 OR S14 OR S15 OR S16 OR S17

S19	TI ( ( ("singl*" OR "doubl*" OR "trebl*" OR "tripl*") N1 ("blind*" OR "mask*") ) ) OR AB ( ( ("singl*" OR "doubl*" OR "trebl*" OR "tripl*") N1 ("blind*" OR "mask*") ) )

S20	TI ( ( "Systematic review*" OR AB "Systematic review*" ) ) OR AB ( ( TI "Systematic review*" OR AB "Systematic review*" ) )

S21	TI ( "randomized controlled trial" OR "controlled clinical trial" OR "placebo*" ) OR AB ( "randomized controlled trial" OR "controlled clinical trial" OR "placebo*" )

S22	TI ( meta-analys* OR metaanalys* OR meta analys* ) OR AB ( meta-analys* OR metaanalys* OR meta analys* )

S23	"controlled trial" OR "clinical trial" or random* or "random sampling" or "clinical trial* or "crossover" OR "crossover" OR "cross over"

S24	"feasibility stud*" OR "pilot project" OR "pilot stud*" OR ( "control and group" ) OR "cohort stud*" OR "comparative stud*"

S25	S19 OR S20 OR S21 OR S22 OR S23 OR S24

S26	S6 AND S18 AND S25
