# Supplementary material for: A Physical Activity Clinical Practice Guideline for People With Moderate to Severe Traumatic Brain Injury
Source: Clin Public Health Guidel. 2025 Aug 14;2(4):e70035. doi: 10.1002/gin2.70035 (PMC13563333; doi:10.1002/gin2.70035)
Supplement: Supplementary file 8 [file GIN2-2-e70035-s008.docx]

**Evidence Profile**

| **Outcome** | **Systematic review evidence  Review credibility** | **Quality Assessment** | | | | | | **Description of evidence  Summary of findings** | **Certainty** | **Explanation** |
| --- | --- | --- | --- | --- | --- | --- | --- | --- | --- | --- |
|  |  | **No. of studies/Study design  No. of participants** | **Risk of bias** | **Inconsistency** | **Indirectness** | **Imprecision** | **Other** |  |  |  |
| Physical  function | Clark 2021  Moderate | 21 RCTs  N= 1412 | No serious  Risk of bias | No serious  inconsistency | No serious indirectness | Serious imprecision | Publication  bias strongly suspected | Mean age ranged from 44 to 76.5 years. Participants included patients both in the acute and chronic phase of stroke. The duration of rehabilitation ranged from two weeks to six months. Minutes of rehabilitation per week ranged from 90 to 1288, with frequency ranging from three to seven days per week. Interventions included physiotherapy, occupational therapy, neuromuscular electrical stimulation and task specific training. Additional time spent in rehabilitation was not associated with improvements in ADL outcome immediately after intervention. There was also no beneficial effect found on activity measures of the upper or lower limb, or motor impairment measures of the supper limb. The review did find a beneficial effect of additional time spent in rehabilitation for motor impairment measures of the lower limb immediately after intervention [SMD 0.71 (95% CI 0.15-1.28); p=0.01; 1 study, n=51; very low-certainty evidence]. | LOW | Certainty of evidence downgraded given, serious imprecision (wide confidence intervals, small sample sizes) and publication bias |
|  | Saunders 2020  Low | 26 RCTs  N-1235 | No serious risk of bias | No serious  inconsistency | No serious indirectness | Serious imprecision | Publication  bias undetected | Cardiorespiratory fitness training interventions ranged from 2 to 24 weeks, session frequency ranged from 2 to 5 days per week, and session duration from 7 to 60 minutes. Interventions included treadmill and overground walking training, cycle ergometry and circuit training.   Two studies found that cardiorespiratory training was beneficial for overall function assessed by the Rivermead Mobility Index (MD 1.56, 95% CI 0,2 to 2.92, p=0.02). However, no effect was found using the Functional Independence Measure and Barthel Index.   Meta-analysis of nine studies (n=317) found training was associated with a significant increase in cardiorespiratory fitness (MD 3.40 mL/ kg/minute, 95% CI 2.98 to 3.83; p = 0.00001).  Training was also associated with improvements in balance (MD 1.92, 95% CI 0.16 to 3.68; P = 0.03, 7 studies, n=471) and functional outcomes as indicated by the Timed Up and Go test (MD −3.42 sec, 95% CI −4.78 to −2.05; P value 0.00001, 5 studies, n=223) and 6 minute walk test (MD +33.41 metres/6 minutes, 95% CI 19.04 to 47.78; P = 0.00001, 16 studies, n=882). | MODERATE | Certainty of evidence downgraded due to serious imprecision (wide confidence intervals) |
|  | Thijs 2023  High | 20 RCTs  N=662 | Serious risk  of bias | Serious  inconsistency | No serious  indirectness | Serious  imprecision | Publication bias  strongly suspected | Mean age in the experimental group ranged from 44 years to 75 years. Mean age in the control group ranged from 48 to 76 years. Total time training ranged from 30 minutes to 45 hours. Interventions included core-stability training, electrical stimulation, selective-trunk training, sitting-reaching therapy, trunk balance training and weight shifting training.   Trunk training was associated with improved activities of daily living when compared to a control group that received non-dose-matched therapy (SMD 0.96 , 95% CI 0.69 to 1.24, p < 0.001, 5 trials, n-283). A positive effect was also found in trunk function (SMD 1.49,95% CI 1.26 to 1.71; P < 0.001; 14 trials, n=466), arm-hand function (SMD 0.67, 95% CI 0.19 to 1.15; P = 0.006; 2 trials, n=74), arm hand activity (SMD 0.84, 95% CI 0.009 to 1.59; P = 0.03; 1 trial, n=30), standing balance (SMD 0.57, 95% CI 0.35 to 0.79; P < 0.001; 11 trials, n=410) leg function (SMD 1.10, 95% CI 0.57 to 1.63; P < 0.001; 1 trial, n=64), and walking ability (SMD 0.73, 95% CI 0.52 to 0.94; P < 0.001; 11 trials, n=383) | VERY LOW | Certainty of evidence downgraded given serious risk of bias, serious inconsistency, serious imprecision and serious publication bias |
| Cognition | Lin 2022  Low | 22 RCTs  N= 1,601 | No serious risk of bias | No serious  inconsistency | No serious  indirectness | No serious  imprecision | Publication  bias strongly suspected | Average age was 63 years. Women made up 40% of the sample. Time from stroke to intervention ranged from <1 week to over 6 years. Physical activity interventions ranged from 4 to 72 weeks, 15-240minuntes per day, 2-5 times per week. Trials investigated aerobic training, strength/balance/stretching/physiotherapy and combined training. Control groups included usual care, PA without a primary aerobic component and additional non-PA intervention.   Physical activity was associated with a significant and positive effect on general cognition [SMD 0.2 (95% CI 0.12-0.27), p<0.001; 22 trials; n=1601]. | MODERATE | Certainty of evidence downgraded given serious indirectness (intervention protocols) |
| Quality of  life | Saunders 2020  Low | 4 RCTs  N=271 | No serious  risk of bias | No serious  inconsistency | No serious  indirectness | Serious  imprecision | Publication  bias  undetected | Mean age ranged from 63 to 73 years in the intervention group and 63 to 70 years in the control group. Training interventions ranged from 8 to 16 weeks, frequency of session ranged from 2-3 times per week and session duration from 15 to 60 minutes. Interventions included overground and treadmill walking and cycling.   Meta-analysis of two studies showed that cardiorespiratory training was associated with a benefit for the 'physical health' component of the SF-12 and SF-36 scale (SMD 0.51, 95% CI 0.2 to 0.82, p=0.001, 2 studies, n=164), but not the 'mental health' component. Two studies showed no pooled effect of training on EuroQoL scores. | MODERATE | Certainty of evidence downgraded given serious imprecision (wide confidence intervals) |
|  | Thijs 2023  High | 2 RCTs  N=108 | Serious risk  of bias | Serious inconsistency | No serious indirectness | Serious  imprecision | None | Mean age ranged from 51 to 60 years in experimental groups, and 63 to 66 years in the control groups. Mean time post-stroke ranged from 51 days to >3 months in the experimental group and 55 days to >3 months in the control group. Total duration ranged from 6-8 weeks, frequency 3-5 times per week, session duration 65-90 minutes. Interventions included neuromuscular electrical stimulation and video-game based therapy.   Trunk training was associated with improved quality of life when compared to a control group that received non-dose-matched therapy (SMD 0.50, 95% CI 0.11 to 0.89; P = 0.01; 2 trials, n=108). | VERY LOW | Certainty of evidence downgraded due to serious risk of bias, serious inconsistency and serious imprecision (small sample size) |
| Mood | Saunders 2020  Low | 2 RCTS  N=56 | Serious risk  of bias | No serious  inconsistency | No serious  indirectness | Serious  imprecision | None | Mean age ranged from 52 to 58 years in the intervention groups and 53 to 56 in the control groups. Physical activity interventions ranged from 4 to 12 weeks, intervention frequent 2-3 times per week and session duration 20 to 60 minutes. Trials investigated aquatic physical training and treadmill training.  The analysis showed no beneficial effect of cardiorespiratory training on mood. | LOW | Certainty of evidence downgraded given serious risk of bias and serious imprecision (small sample size) |

**Systematic Reviews Data Extraction**

| **Systematic Review and Meta-Analysis**  **Citation**: Clark B, Whitall J, Kwakkel G, Mehrholz J, Ewings S, Burridge J. The effect of time spent in rehabilitation on activity limitation and impairment after stroke. *Cochrane Database Syst Rev*. 2021; 10(10): CD012612. | |
| --- | --- |
| **Purpose:** To assess the effect of 1. more time spent in the same type of rehabilitation on activity measures in people with stroke; 2. difference in total rehabilitation time (in minutes) on recovery of activity in people with stroke; and 3. rehabilitation schedule on activity in terms of: a. average time (minutes) per week undergoing rehabilitation, b. frequency (number of sessions per week) of rehabilitation, and c. total duration of rehabilitation. | **Background:** Stroke affects millions of people every year and is a leading cause of disability, resulting in significant financial cost and reduction in quality of life. Rehabilitation after stroke aims to reduce disability by facilitating recovery of impairment, activity, or participation. One aspect of stroke rehabilitation that may affect outcomes is the amount of time spent in rehabilitation, including minutes provided, frequency (i.e. days per week of rehabilitation), and duration (i.e. time period over which rehabilitation is provided). Effect of time spent in rehabilitation after stroke has been explored extensively in the literature, but findings are inconsistent. Previous systematic reviews with meta-analyses have included studies that differ not only in the amount provided, but also type of rehabilitation.  **Objectives:** To assess the effect of 1. more time spent in the same type of rehabilitation on activity measures in people with stroke; 2. difference in total rehabilitation time (in minutes) on recovery of activity in people with stroke; and 3. rehabilitation schedule on activity in terms of: a. average time (minutes) per week undergoing rehabilitation, b. frequency (number of sessions per week) of rehabilitation, and c. total duration of rehabilitation.  **Search methods:** We searched the Cochrane Stroke Group trials register, CENTRAL, MEDLINE, Embase, eight other databases, and five trials registers to June 2021. We searched reference lists of identified studies, contacted key authors, and undertook reference searching using Web of Science Cited Reference Search.  **Selection criteria:** We included randomised controlled trials (RCTs) of adults with stroke that compared different amounts of time spent, greater than zero, in rehabilitation (any non-pharmacological, non-surgical intervention aimed to improve activity after stroke). Studies varied only in the amount of time in rehabilitation between experimental and control conditions. Primary outcome was activities of daily living (ADLs); secondary outcomes were activity measures of upper and lower limbs, motor impairment measures of upper and lower limbs, and serious adverse events (SAE)/death.  **Data collection and analysis:** Two review authors independently screened studies, extracted data, assessed methodological quality using the Cochrane RoB 2 tool, and assessed certainty of the evidence using GRADE. For continuous outcomes using different scales, we calculated pooled standardised mean difference (SMDs) and 95% confidence intervals (CIs). We expressed dichotomous outcomes as risk ratios (RR) with 95% CIs.  **Main results:** The quantitative synthesis of this review comprised 21 parallel RCTs, involving analysed data from 1412 participants. Time in rehabilitation varied between studies. Minutes provided per week were 90 to 1288. Days per week of rehabilitation were three to seven. Duration of rehabilitation was two weeks to six months. Thirteen studies provided upper limb rehabilitation, five general rehabilitation, two mobilisation training, and one lower limb training. Sixteen studies examined participants in the first six months following stroke; the remaining five included participants more than six months poststroke. Comparison of stroke severity or level of impairment was limited due to variations in measurement. The risk of bias assessment suggests there were issues with the methodological quality of the included studies. There were 76 outcome-level risk of bias assessments: 15 low risk, 37 some concerns, and 24 high risk. When comparing groups that spent more time versus less time in rehabilitation immediately after intervention, we found no difference in rehabilitation for ADL outcomes (SMD 0.13, 95% CI -0.02 to 0.28; P = 0.09; I2 = 7%; 14 studies, 864 participants; very low-certainty evidence), activity measures of the upper limb (SMD 0.09, 95% CI -0.11 to 0.29; P = 0.36; I2 = 0%; 12 studies, 426 participants; very low-certainty evidence), and activity measures of the lower limb (SMD 0.25, 95% CI -0.03 to 0.53; P = 0.08; I2 = 48%; 5 studies, 425 participants; very low-certainty evidence). We found an effect in favour of more time in rehabilitation for motor impairment measures of the upper limb (SMD 0.32, 95% CI 0.06 to 0.58; P = 0.01; I2 = 10%; 9 studies, 287 participants; low-certainty evidence) and of the lower limb (SMD 0.71, 95% CI 0.15 to 1.28; P = 0.01; 1 study, 51 participants; very low-certainty evidence). There were no intervention-related SAEs. More time in rehabilitation did not affect the risk of SAEs/death (RR 1.20, 95% CI 0.51 to 2.85; P = 0.68; I2 = 0%; 2 studies, 379 participants; low-certainty evidence), but few studies measured these outcomes. Predefined subgroup analyses comparing studies with a larger difference of total time spent in rehabilitation between intervention groups to studies with a smaller difference found greater improvements for studies with a larger difference. This was statistically significant for ADL outcomes (P = 0.02) and activity measures of the upper limb (P = 0.04), but not for activity measures of the lower limb (P = 0.41) or motor impairment measures of the upper limb (P = 0.06).  **Authors'** **conclusions:** An increase in time spent in the same type of rehabilitation after stroke results in little to no difference in meaningful activities such as activities of daily living and activities of the upper and lower limb but a small benefit in measures of motor impairment (low- to very low-certainty evidence for all findings). If the increase in time spent in rehabilitation exceeds a threshold, this may lead to improved outcomes. There is currently insufficient evidence to recommend a minimum beneficial daily amount in clinical practice. The findings of this study are limited by a lack of studies with a significant contrast in amount of additional rehabilitation provided between control and intervention groups. Large, well-designed, high-quality RCTs that measure time spent in all rehabilitation activities (not just interventional) and provide a large contrast (minimum of 1000 minutes) in amount of rehabilitation between groups would provide further evidence for effect of time spent in rehabilitation. |
| **Timeframe:** Inception - June 2021 |  |
| **Total # studies**  **included:** 21 |  |
| **Other details (e.g. definitions used, exclusions etc)**  Participants were adults (aged over 18 years), with a clinical diagnosis of stroke, caused by either infarct or haemorrhage (including subarachnoid haemorrhage), as defined by the study authors. Participants received rehabilitation in an inpatient, outpatient, or community setting. We excluded studies that included participants with diagnoses other than stroke as the primary diagnosis, even if they included some participants with a primary diagnosis of stroke.  Comparisons of intervention versus no intervention (including trials in which just some participants received no intervention), were excluded. |  |
| **Outcomes**  **addressed:** Physical function |  |

| **Systematic Review and Meta-Analysis**  **Citation**: Saunders DH, Sanderson M, Hayes S, Johnson L, Kramer S, Carter DD, Jarvis H, Brazzelli M, Mead GE. Physical fitness training for stroke patients. *Cochrane Database Syst Rev.* 2020; Issue 3. Art. No: CD003316. | |
| --- | --- |
| **Purpose:** To determine whether fitness training after stroke reduces death, death or dependence, and disability. The secondary objectives were to determine the effects of training on adverse events, risk factors, physical fitness, mobility, physical function, health status and quality of life, mood, and cognitive function. | **Background:** Levels of physical activity and physical fitness are low after stroke. Interventions to increase physical fitness could reduce mortality and reduce disability through increased function.  **Objectives:** The primary objectives of this updated review were to determine whether fitness training after stroke reduces death, death or dependence, and disability. The secondary objectives were to determine the effects of training on adverse events, risk factors, physical fitness, mobility, physical function, health status and quality of life, mood, and cognitive function.  **Search methods:** In July 2018 we searched the Cochrane Stroke Trials Register, CENTRAL, MEDLINE, Embase, CINAHL, SPORTDiscus, PsycINFO, and four additional databases. We also searched ongoing trials registers and conference proceedings, screened reference lists, and contacted experts in the field.  **Selection criteria:** Randomised trials comparing either cardiorespiratory training or resistance training, or both (mixed training), with usual care, no intervention, or a non-exercise intervention in stroke survivors.  **Data collection and analysis:** Two review authors independently selected studies, assessed quality and risk of bias, and extracted data. We analysed data using random-effects meta-analyses and assessed the quality of the evidence using the GRADE approach. Diverse outcome measures limited the intended analyses.  **Main results:** We included 75 studies, involving 3017 mostly ambulatory participants, which comprised cardiorespiratory (32 studies, 1631 participants), resistance (20 studies, 779 participants), and mixed training interventions (23 studies, 1207 participants). Death was not influenced by any intervention; risk differences were all 0.00 (low-certainty evidence). There were few deaths overall (19/3017 at end of intervention and 19/1469 at end of follow-up). None of the studies assessed death or dependence as a composite-outcome. Disability scores were improved at end of intervention by cardiorespiratory training (standardised mean difference (SMD) 0.52,95% CI 0.19 to 0.84; 8 studies, 462 participants; P = 0.002; moderate-certainty evidence) and mixed training (SMD 0.23, 95% CI 0.03 to 0.42; 9studies, 604 participants; P = 0.02; low-certainty evidence). There were too few data to assess the effects of resistance training on disability. Secondary outcomes showed multiple benefits for physical fitness (VO2 peak and strength), mobility (walking speed) and physical function(balance). These physical effects tended to be intervention-specific with the evidence mostly low or moderate certainty. Risk factor data were limited or showed no effects apart from cardiorespiratory fitness (VO2 peak), which increased after cardiorespiratory training (mean difference (MD) 3.40 mL/kg/min, 95% CI 2.98 to 3.83; 9 studies, 438 participants; moderate-certainty evidence). There was no evidence of any serious adverse events. Lack of data prevents conclusions about effects of training on mood, quality of life, and cognition. Lack of data also meant benefits at follow-up (i.e., after training had stopped) were unclear but some mobility benefits did persist. Risk of bias varied across studies but imbalanced amounts of exposure in control and intervention groups was a common issue affecting many comparisons.  **Authors'** **conclusions:** Few deaths overall suggest exercise is a safe intervention but means we cannot determine whether exercise reduces mortality or the chance of death or dependency. Cardiorespiratory training and, to a lesser extent mixed training, reduce disability during or after usual stroke care; this could be mediated by improved mobility and balance. There is sufficient evidence to incorporate cardiorespiratory and mixed training, involving walking, within post-stroke rehabilitation programmes to improve fitness, balance and the speed and capacity of walking. The magnitude of VO2 peak increase after cardiorespiratory training has been suggested to reduce risk of stroke hospitalisation by ˜7%. Cognitive function is under-investigated despite being a key outcome of interest for patients. Further well-designed randomised trials are needed to determine the optimal exercise prescription, the range of benefits and any long-term benefits. |
| **Timeframe:** Inception - July 2018 |  |
| **Total # studies**  **included:** 75 |  |
| **Other details (e.g. definitions used, exclusions etc)**  Studies that focused on different types of standard rehabilitation techniques but did not include a physical fitness training component were excluded. Studies that combined fitness training with assistive technologies, such as robotic and electromechanical-assisted gait training devices during body weight-supported locomotor training, as well as studies investigating virtual reality approaches were excluded. Studies that compared upper and lower body training were excluded if an additional non-exercise control group was not considered. Comparisons of intervention versus no intervention (including trials in which just some participants received no intervention), were excluded. |  |
| **Outcomes**  **addressed:** Physical function, quality of life |  |

| **Systematic Review and Meta-Analysis**  **Citation**: Thijs L, Voets E, Denissen S, Mehrholz J, Elsner B, Lemmens R, Verheyden GS. Trunk training following stroke. *Cochrane Database Syst Rev.* 2020; Issue 3. Art. No: CD013712. | |
| --- | --- |
| **Purpose:** To assess the effectiveness of trunk training after stroke on activities of daily living (ADL), trunk function, arm-hand function or activity, standing balance, leg function, walking ability, and quality of life when comparing with both dose-matched as non-dose-matched control groups | **Background:** Previous systematic reviews and randomised controlled trials have investigated the effect of post-stroke trunk training. Findings suggest that trunk training improves trunk function and activity or the execution of a task or action by an individual. But it is unclear what effect trunk training has on daily life activities, quality of life, and other outcomes.  **Objectives:** To assess the effectiveness of trunk training after stroke on activities of daily living (ADL), trunk function, arm-hand function or activity, standing balance, leg function, walking ability, and quality of life when comparing with both dose-matched as non-dose-matched control groups  **Search methods:** We searched the Cochrane Stroke Group Trials Register, CENTRAL, MEDLINE, Embase, and five other databases to 25 October 2021. We searched trial registries to identify additional relevant published, unpublished, and ongoing trials. We hand searched the bibliographies of included studies.  **Selection criteria:** We selected randomised controlled trials comparing trunk training versus non-dose-matched or dose-matched control therapy including adults (18 years or older) with either ischaemic or haemorrhagic stroke. Outcome measures of trials included ADL, trunk function, arm-hand function or activity, standing balance, leg function, walking ability, and quality of life.  **Data collection and analysis:** We used standard methodological procedures expected by Cochrane. Two main analyses were carried out. The first analysis included trials where the therapy duration of control intervention was non-dose matched with the therapy duration of the experimental group and the second analysis where there was comparison with a dose-matched control intervention (equal therapy duration in both the control as in the experimental group).  **Main results:** We included 68 trials with a total of 2585 participants. In the analysis of the non-dose-matched groups (pooling of all trials with different training duration in the experimental as in the control intervention), we could see that trunk training had a positive effect on ADL (standardised mean difference (SMD) 0.96; 95% confidence interval (CI) 0.69 to 1.24; P < 0.001; 5 trials; 283 participants; very low-certainty evidence), trunk function (SMD 1.49, 95% CI 1.26 to 1.71;P < 0.001; 14 trials, 466 participants; very low-certainty evidence), arm-hand function (SMD 0.67, 95% CI 0.19 to 1.15; P = 0.006; 2 trials, 74 participants; low-certainty evidence), arm-hand activity (SMD 0.84, 95% CI 0.009 to 1.59; P = 0.03; 1 trial, 30 participants; very low-certainty evidence), standing balance (SMD 0.57, 95% CI 0.35 to 0.79; P < 0.001; 11 trials, 410 participants; very low-certainty evidence), leg function (SMD 1.10, 95% CI 0.57 to 1.63; P < 0.001; 1 trial, 64 participants; very low-certainty evidence), walking ability (SMD 0.73, 95% CI 0.52 to0.94; P < 0.001; 11 trials, 383 participants; low-certainty evidence) and quality of life (SMD 0.50, 95% CI 0.11 to 0.89; P = 0.01; 2 trials, 108 participants; low-certainty evidence). Non-dose-matched trunk training led to no difference for the outcome serious adverse events (odds ratio: 7.94, 95% CI 0.16 to 400.89; 6 trials, 201 participants; very low-certainty evidence).  In the analysis of the dose-matched groups (pooling of all trials with equal training duration in the experimental as in the control intervention), we saw that trunk training had a positive effect on trunk function (SMD 1.03, 95% CI 0.91 to 1.16; P < 0.001; 36 trials, 1217participants; very low-certainty evidence), standing balance (SMD 1.00, 95% CI 0.86 to 1.15; P < 0.001; 22 trials, 917 participants; very low-certainty evidence), leg function (SMD 1.57, 95% CI 1.28 to 1.87; P < 0.001; 4 trials, 254 participants; very low-certainty evidence), walking ability (SMD 0.69, 95% CI 0.51 to 0.87; P < 0.001; 19 trials, 535 participants; low-certainty evidence) and quality of life (SMD 0.70, 95% CI0.29 to 1.11; P < 0.001; 2 trials, 111 participants; low-certainty evidence), but not for ADL (SMD 0.10; 95% confidence interval (CI) -0.17 to0.37; P = 0.48; 9 trials; 229 participants; very low-certainty evidence), arm-hand function (SMD 0.76, 95% CI -0.18 to 1.70; P = 0.11; 1 trial,19 participants; low-certainty evidence), arm-hand activity (SMD 0.17, 95% CI -0.21 to 0.56; P = 0.38; 3 trials, 112 participants; very low-certainty evidence). Trunk training also led to no difference for the outcome serious adverse events (odds ratio (OR): 7.39, 95% CI 0.15 to372.38; 10 trials, 381 participants; very low-certainty evidence).  Time post stroke led to a significant subgroup difference for standing balance (P < 0.001) in non-dose-matched therapy. In non-dose-matched therapy, different trunk therapy approaches had a significant effect on ADL (< 0.001), trunk function (P < 0.001) and standing balance (< 0.001).When participants received dose-matched therapy, analysis of subgroup differences showed that the trunk therapy approach had a significant effect on ADL (P = 0.001), trunk function (P < 0.001), arm-hand activity (P < 0.001), standing balance (P = 0.002), and leg function(P = 0.002).  Also, for dose-matched therapy, subgroup analysis for time post stroke resulted in a significant difference for the outcomes standing balance (P < 0.001), walking ability (P = 0.003) and leg function (P < 0.001), time post stroke significantly modified the effect of intervention. Core-stability trunk (15 trials), selective-trunk (14 trials) and unstable-trunk (16 trials) training approaches were mostly applied in the included trials.  **Authors'** **conclusions:** There is evidence to suggest that trunk training as part of rehabilitation improves ADL, trunk function, standing balance, walking ability, upper and lower limb function, and quality of life in people after stroke. Core-stability, selective-, and unstable-trunk training were the trunk training approaches mostly applied in the included trials. When considering only trials with a low risk of bias, results were mostly confirmed, with very low to moderate certainty, depending on the outcome. |
| **Timeframe:** Inception - October 2021 |  |
| **Total # studies**  **included:** 68 |  |
| **Other details (e.g. definitions used, exclusions etc)**  Trials including other diseases in addition to stroke were excluded, unless they reported separate results for the stroke participants of interest.  Cross-over RCTs were not included. If trunk training was embedded in a broader training concept, such as circuit training or a general strength programme, this study was excluded from this review |  |
| **Outcomes**  **addressed:** Physical function, quality of life |  |

| **Systematic Review and Meta-Analysis**  **Citation**: Lin H, Liu HH, Dai Y, Yin X, Li Z, Yang L, Tao J, Liu W, Chen L. Effect of physical activity on cognitive impairment in patients with cerebrovascular diseases: a systematic review and meta-analysis. *Front Neurol.* 2022; 13: 854158. | |
| --- | --- |
| **Purpose:** This study investigates the effect of physical activity (PA) on cognition in patients with cerebrovascular disease and explored the maximum benefit of different PA characteristics. | **Background and Purpose:** This study investigates the effect of physical activity (PA) on cognition in patients with cerebrovascular disease and explored the maximum benefit of different PA characteristics.  **Methods:** Databases, such as Pubmed, Web of Science, Embase, and Cochrane Library, were searched from their inception to May 31, 2021. Standardized mean difference (SMD) and 95% confidence intervals (CIs) were calculated to generate a forest plot. In addition, subgroup analysis, moderation analysis, and regression analysis were performed to explore the possible adjustment factors.  **Results:** In total, 22 studies that met the criteria were included, demonstrating data from 1,601 participants. The results indicated that PA produced a positive effect on the global cognition for patients with cerebrovascular disease (SMD: 0.20 [95% CI: 0.12–0.27]), at the same time, PA training prominently improved executive function (SMD: 0.09 [95% CI: 0.00–0.17]) and working memory (SMD: 0.25 [95% CI: 0.10–0.40]). Furthermore, patients with baseline cognitive impairment received the greater benefit of PA on cognition (SMD: 0.24 [95% CI: 0.14–0.34]) than those without cognitive impairment before intervention (SMD: 0.15 [95% CI: 0.04–0.26]). For patients in the acute stage (≤ 3 months), PA did not rescue impairment dysfunction significantly (SMD: 0.08 [95% CI: −0.04–0.21]) and remarkable cognitive gains were detected in the chronic stage of participants (>3 months) (SMD: 0.25 [95% CI: 0.16–0.35]). Moderate intensity PA showed a larger pooled effect size (SMD: 0.23 [95% CI: 0.11–0.36]) than low intensity (SMD: −0.01 [95% CI: −0.44–0.43]) and high intensity (SMD: 0.16 [95% CI: 0.03–0.29]). However, the different types, duration, and frequency of PA resulted in no differences in the improvement of cognitive function. Further regression analysis demonstrated that the beneficial effects of PA on cognition are negatively correlated with age (p < 0.05)  Time post stroke led to a significant subgroup difference for standing balance (P < 0.001) in non-dose-matched therapy. In non-dose-matched therapy, different trunk therapy approaches had a significant effect on ADL (< 0.001), trunk function (P < 0.001) and standing balance (< 0.001).When participants received dose-matched therapy, analysis of subgroup differences showed that the trunk therapy approach had a significant effect on ADL (P = 0.001), trunk function (P < 0.001), arm-hand activity (P < 0.001), standing balance (P = 0.002), and leg function(P = 0.002).  **Conclusions:** This study revealed that PA can prominently improve the cognitive ability in patients with cerebrovascular diseases and strengthened the evidence that PA held promise as a widely accessible and effective non-drug therapy for vascular cognitive impairment (VCI). |
| **Timeframe:** Inception - May 2021 |  |
| **Total # studies**  **included:** 22 |  |
| **Other details (e.g. definitions used, exclusions etc)**  Included subjects cannot have other neurodegenerative diseases or serious mental diseases that can cause cognitive impairment |  |
| **Outcomes**  **addressed:** Cognition |  |

**AMSTAR 2 Ratings**

| Author, Year | PICO^1^ | A priori Methods^2^ | Study Design Selection^3^ | Search Strategy^4^ | Study Selection^5^ | Data Extraction^6^ | Excluded Studies^7^ | Included Studies^8^ | RoB Assessment^9^ | Funding Sources^10^ | Statistical Methods^11^ | Impact of RoB^12^ | RoB Results^13^ | Heterogeneity^14^ | Publication Bias^15^ | COI^16^ | Overall Rating |
| --- | --- | --- | --- | --- | --- | --- | --- | --- | --- | --- | --- | --- | --- | --- | --- | --- | --- |
| Abba, 2022 | Y | N | N | N | N | N | N | N | PY | N | N/A | N/A | Y | Y | N/A | Y | Critically low |
| Amorós-Aguilar, 2021 | Y | N | N | N | Y | Y | N | PY | Y | N | N/A | N/A | Y | Y | N/A | Y | Critically low |
| Anjos, 2022 | Y | PY | N | PY | Y | Y | N | N | N | N | N | N | Y | N | N | Y | Critically low |
| Barclay, 2022 | N | N | N | N | N | N | N | Y | PY | N | Y | N | N | Y | N | Y | Critically low |
| Bressi, 2022 | N | PY | N | N | Y | N | N | PY | Y | Y | N/A | N/A | N | N | N/A | Y | Critically low |
| Cai, 2022 | Y | Y | N | N | Y | Y | N | N | Y | N | Y | Y | Y | Y | Y | Y | Critically low |
| Chen, 2022 | Y | Y | N | N | Y | Y | N | PY | Y | N | N | Y | Y | Y | Y | Y | Critically low |
| Chiaramonte, 2022 | Y | PY | N | N | Y | Y | N | PY | Y | N | N/A | N/A | Y | Y | N/A | Y | Critically low |
| Clark, 2021 | Y | Y | Y | Y | N | Y | Y | PY | Y | Y | Y | N | Y | Y | Y | Y | Moderate |
| Cronin, 2023 | Y | PY | Y | N | N | N | N | PY | PY | N | N | N | N | N | N | Y | Critically low |
| Giuriati, 2021 | N | N | N | N | N | N | N | PY | PY | N | N | N | N | N | N | N | Critically low |
| Khattab, 2021 | Y | PY | N | N | Y | Y | N | PY | Y | N | Y | N | N | Y | N | Y | Critically low |
| Lenoir, 2021 | Y | N | Y | PY | Y | Y | N | PY | Y | N | N/A | N/A | Y | Y | N/A | Y | Critically low |
| Li, 2022 | Y | N | N | N | N | Y | N | N | Y | N | N | N | N | N | N | Y | Critically low |
| Li, 2023 | Y | PY | N | N | N | Y | N | PY | Y | N | N | N | N | N | N | Y | Critically low |
| Lin, 2022 | Y | PY | N | PY | N | Y | N | N | Y | N | Y | N | Y | N | Y | Y | Low |
| Lyu, 2021 | Y | Y | N | N | Y | Y | N | N | Y | Y | Y | N | Y | N | N | Y | Critically low |
| Mayer, 2021 | N | N | N | N | N | Y | N | N | PY | N | N/A | N/A | Y | N | N/A | Y | Critically low |
| Nindorera, 2022 | Y | Y | N | N | Y | Y | N | PY | Y | N | Y | Y | N | N | N | Y | Critically low |
| Perez-Rodriguez, 2022 | N | PY | N | PY | Y | N | N | N | Y | N | N | N | N | Y | N | Y | Critically low |
| Postol, 2019 | Y | PY | N | N | N | N | N | PY | PY | N | N | N | Y | N | N | N | Critically low |
| Rintala, 2022 | Y | N | N | N | Y | N | N | Y | PY | N | N/A | N/A | N | Y | N/A | Y | Critically low |
| Saquetto, 2019 | Y | N | N | PY | Y | Y | N | N | PY | N | N | N | N | N | N | Y | Critically low |
| Saunders, 2020 | Y | PY | N | Y | Y | N | Y | Y | Y | Y | Y | N | N | N | Y | Y | Low |
| Shu, 2020 | Y | N | N | N | N | N | N | PY | Y | N | N | N | N | N | N | Y | Critically low |
| Sun, 2021 | Y | PY | N | N | Y | N | N | N | Y | N | N | N | Y | N | N | Y | Critically low |
| Tai, 2022 | N | PY | N | N | Y | Y | N | PY | Y | N | N/A | N/A | Y | Y | N/A | Y | Critically low |
| Thijs, 2023 | Y | Y | N | Y | Y | Y | Y | PY | Y | Y | Y | Y | Y | Y | Y | Y | High |
| Veldema, 2020 (RT) | Y | N | N | N | Y | N | N | PY | PY | N | N | N | Y | Y | N | Y | Critically low |
| Veldema, 2020 (AT) | N | N | N | N | N | N | N | PY | PY | N | N | N | N | N | N | N | Critically low |
| Veldema, 2021 | Y | N | N | N | N | N | N | PY | PY | N | N | N | Y | Y | N | Y | Critically low |
| Walter, 2022 | N | N | N | N | N | N | N | N | Y | N | N/A | N/A | N | Y | N/A | Y | Critically low |
| Zhang, 2023 | Y | PY | Y | N | Y | Y | N | PY | Y | N | N | N | Y | N | Y | Y | Critically low |
| Zheng, 2021 | Y | Y | N | PY | Y | Y | N | PY | Y | Y | N | N | Y | N | N | Y | Critically low |
| Zhou, 2022 | N | PY | N | N | Y | Y | N | PY | Y | N | Y | N | Y | Y | Y | Y | Critically low |

PICO, Participant, Intervention, Comparator, Outcome; ROB, Risk of Bias; COI, Conflict of Interest; Y, Yes; N, No; PY, Partially Yes; N/A, Not Applicable.

^1^ Did the research questions and inclusion criteria for the review include the components of PICO?

^2^ Did the report of the review contain an explicit statement that the review methods were established prior to the conduct of the review and did the report justify any significant deviations from the protocol?

^3^ Did the review authors explain their selection of the study designs for inclusion in the review?

^4^ Did the review authors use a comprehensive literature search strategy?

^5^ Did the review authors perform study selection in duplicate?

^6^ Did the review authors perform data extraction in duplicate?

^7^ Did the review authors provide a list of excluded studies and justify the exclusions?

^8^ Did the review authors describe the included studies in adequate detail?

^9^ Did the review authors use a satisfactory technique for assessing the RoB in individual studies that were included in the review?

^10^ Did the review authors report on the sources of funding for the studies included in the review?

^11^ If meta-analysis was performed did the review authors use appropriate methods for statistical combination of results?

^12^ If meta-analysis was performed, did the review authors assess the potential impact of RoB in individual studies on the results of the meta-analysis or other evidence synthesis?

^13^ Did the review authors account for RoB in individual studies when interpreting/ discussing the results of the review?

^14^ Did the review authors provide a satisfactory explanation for, and discussion of, any heterogeneity observed in the results of the review?

^15^ If they performed quantitative synthesis did the review authors carry out an adequate investigation of publication bias (small study bias) and discuss its likely impact on the results of the review?

^16^ Did the review authors report any potential sources of conflict of interest, including any funding they received for conducting the review?
